# POP1 Facilitates Proliferation in Triple-Negative Breast Cancer via m6A-Dependent Degradation of CDKN1A mRNA

**DOI:** 10.34133/research.0472

**Published:** 2024-09-12

**Authors:** Chao Zhang, Sifen Wang, Xiuqing Lu, Wenjing Zhong, Yunyun Tang, Weiling Huang, Fengjia Wu, Xiumei Wang, Weidong Wei, Hailin Tang

**Affiliations:** ^1^ State Key Laboratory of Oncology in South China, Guangdong Provincial Clinical Research Center for Cancer, Sun Yat-sen University Cancer Center, Guangzhou, China.; ^2^ Department of Breast Surgery, Zhongshan City People’s Hospital, ZhongShan, China.; ^3^ Department of Biomedical and Clinical Sciences (BKV), Linköping University, Linköping, Sweden.; ^4^ Guangzhou Kangda Vocational Technical College, Guangzhou 510700, China.; ^5^ Affiliated Cancer Hospital of Inner Mongolia Medical University, Hohhot 010020, Inner Mongolia, China.

## Abstract

Triple-negative breast cancer (TNBC) is currently the worst prognostic subtype of breast cancer, and there is no effective treatment other than chemotherapy. Processing of precursors 1 (POP1) is the most substantially up-regulated RNA-binding protein (RBP) in TNBC. However, the role of POP1 in TNBC remains clarified. A series of molecular biological experiments in vitro and in vivo and clinical correlation analyses were conducted to clarify the biological function and regulatory mechanism of POP1 in TNBC. Here, we identified that POP1 is significantly up-regulated in TNBC and associated with poor prognosis. We further demonstrate that POP1 promotes the cell cycle and proliferation of TNBC in vitro and vivo. Mechanistically, POP1 directly binds to the coding sequence (CDS) region of CDKN1A mRNA and degrades it. The degradation process depends on the N6-methyladenosine (m6A) modification at the 497th site of CDKN1A and the recognition of this modification by YTH N6-methyladenosine RNA binding protein 2 (YTHDF2). Moreover, the m6A inhibitor STM2457 potently impaired the proliferation of POP1-overexpressed TNBC cells and improved the sensitivity to paclitaxel. In summary, our findings reveal the pivotal role of POP1 in promoting TNBC proliferation by degrading the mRNA of CDKN1A and that inhibition of m6A with STM2457 is a promising therapeutic strategy for TNBC.

## Introduction

Triple-negative breast cancer (TNBC) is widely known as the absence of estrogen receptor (ER), progesterone receptor (PR), and human epidermal growth factor receptor-2 (HER2) expressions, which makes it a lack of targets for precise and effective treatment [1,2]. Because of its high proliferative capacity and high aggressiveness, TNBC is the worst prognostic subtype of breast cancer [3]. It accounts for only 15 to 20% of all cases but more than 80% of breast cancer deaths [4]. Uncontrolled proliferation is one of the most prominent features of all malignant tumors, and TNBC is no exception [5,6]. Compared to other subtypes, TNBC often exhibits a higher proliferative capacity, as indicated by the high positivity rate of the proliferative antigen Ki-67 in the tumor tissue of most patients [7–9]. This is also an important reason why it progresses faster and metastasizes earlier [10]. However, the molecular regulatory mechanism of TNBC hyperproliferation is not completely clear to date.

N6-methyladenosine (m6A) is one of the most universal posttranscriptional modifications in mammalian cells and has been proven to play key regulatory roles in most biological processes [11,12]. A range of m6A regulators, including writers, readers, and erasers precisely regulate the stability, localization, and expression of RNAs by adding, removing, and recognizing m6A modifications within the cell [13,14]. Among them, the recognition of m6A is a highly diverse regulatory process. Through different readers, RNAs with m6A modifications at specific sites are directed to different downstream elements, thus mediating a series of biological effects [15]. Therefore, elucidating these direct regulators of m6A along with complex downstream components is of great significance for enhancing the realization of malignant phenotypic regulatory signals and seeking new potential therapeutic targets.

Processing of precursors 1 (POP1), also called ribonuclease (RNase) P/MRP subunit, is an RNase that mainly localizes in the nucleus and acts in pre-RNA processing. It was first reported in yeast as a key protein component of RNase P and RNase MRP involved in posttranscriptional processing of 5.8s rRNA in the 1990s [16]. Subsequent studies were mainly carried out in lower organisms including yeast and *Caenorhabditis elegans*, involving spindle separation regulation and maintenance of telomerase activity [17–19]. As a typical RNA-binding protein (RBP), the biological function of POP1 in human has been poorly understood until Park et al.’s [20] study suggested that RNase P/MRP could cleavage RNAs in an m6A-dependent manner and lead to down-regulation of the corresponding gene. Previous bioinformatics analyses suggested that POP1 is up-regulated in a variety of malignancies, including TNBC, and is correlated with poor prognosis [21]. As a key protein with catalytic activity in RNase P/MRP, its role in tumors needs further investigation.

In this study, we identified that POP1 is up-regulated in TNBC and related to poor prognosis. Further analyses and experiments showed that POP1 promoted cell cycle and proliferation of TNBC by directly binding and degrading CDKN1A mRNA. Besides, this process is highly dependent on the m6A modification of CDKN1A mRNA and the recognition of the reader molecule YTH N6-methyladenosine RNA binding protein 2 (YTHDF2). More importantly, we illustrated that STM2457, an m6A inhibitor, significantly impaired the proliferation mediated by high expression of POP1 and improved the sensitivity of TNBC to the period-specific chemotherapy drug paclitaxel. Overall, our findings partially shed light on a potential regulatory mechanism for the high proliferative capacity of TNBC and suggest a new therapeutic strategy for TNBC.

## Results

### POP1 is up-regulated in TNBC and correlated with poor prognosis

**Fig. 1.**
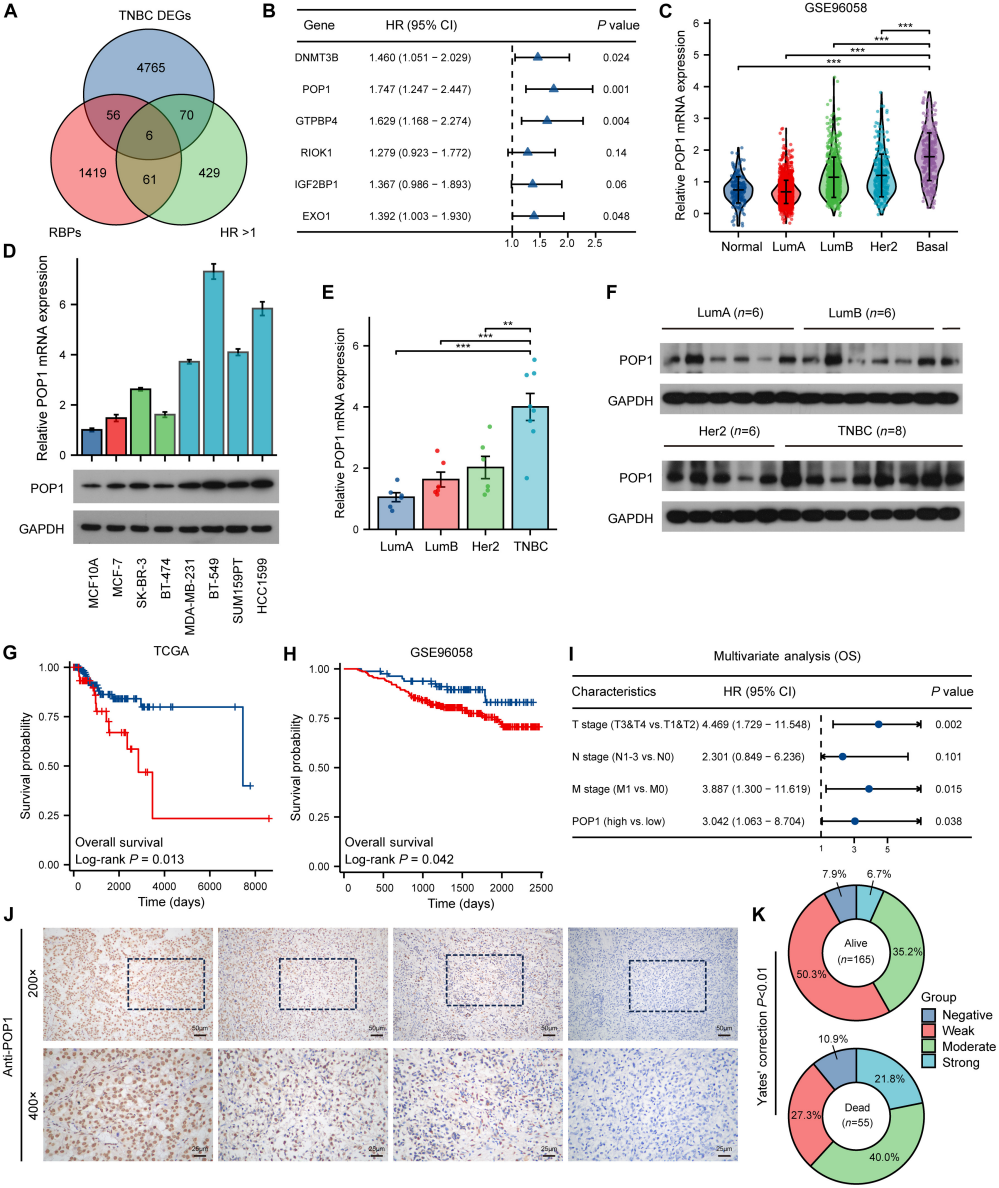
POP1 is up-regulated in TNBC and correlated with poor prognosis. (A) Venn diagram to obtain the gene intersection of differentially expressed genes, RBPs, and hazard ratio (HR) > 1 in TNBC. (B) Univariate Cox regression forest map of 6 RBPs. (C) mRNA expression levels of POP1 in different types of breast cancer tissues and normal breast tissues based on the GSE96058 dataset. (D) Quantitative reverse transcription PCR analysis and Western blot analysis to detect the mRNA and protein expression level of POP1 in different types of breast cancer cell lines and normal breast epithelial cells. The mRNA (E) and protein (F) expression levels of POP1 in different subtypes of breast cancer tissues. Analysis of mRNA levels was achieved using one-way ANOVA based on normality and homogeneity of variance. OS analysis of patients stratified by POP1 expression based on log-rank test in TNBC of TCGA (G) and GSE96058 (H). (I) Multivariate Cox regression forest map based on TNBC data from TCGA. (J) Representative microscopic IHC images of POP1 expression in the tissue sections of TNBC patients. (K) Distribution of POP1 expression in 220 patients in survival and death groups, analyzed by Yates’ correction method. **P < 0.01, ***P < 0.001.

Through a series of screenings, we identified genes that are specifically high expressing in TNBC and are associated with poor prognosis (Fig. S1A to D). Among them, 6 RBPs are worthy of attention, namely, DNMT3B, POP1, GTPBP4, RIOK1, IGF2BP1, and EXO1 (Fig. 1A). We then performed a univariate Cox regression analysis and found that POP1 had the most significant *P* value (Fig. 1B). Two independent datasets, GSE96058 (https://www.ncbi.nlm.nih.gov/geo/query/acc.cgi?acc=gse96058) [22] and TCGA (https://portal.gdc.cancer.gov/) [23], indicated high expression of POP1 in TNBC (Fig. 1C and Fig. S1E). Receiver operating characteristic (ROC) curves indicated that high POP1 expression was effective in the diagnosis of TNBC [area under the curve (AUC) = 0.911, 0.798] (Fig. S1F and G). By examining mRNA and protein expression levels of POP1 in 8 breast cancer cell lines containing different subtypes and tissue samples from 26 patients, we determined that POP1 was significantly up-regulated in TNBC (Fig. 1D to F). *K*–*m* plots suggested that patients with high POP1 expression had a poorer prognosis than those with low POP1 expression (Fig. 1G and H). Further multivariate Cox regression analysis confirmed that POP1 expression along with T stage and M stage were independent predictive factors of TNBC prognosis (Fig. 1I). In addition, we also detected POP1 expression in tissues of 220 TNBC patients diagnosed and treated between 2001 and 2012 in our center by immunohistochemistry (IHC) (Fig. 1J). The results showed that the proportion of patients with high POP1 expression was significantly higher in the 55 patients who died than in the 165 patients who survived. Patients with high or low POP1 expression were grouped and analyzed for survival. Consistent with the results of above analyses, patients with high POP1 expression had worse overall survival (OS) (Fig. 1K). These results suggest that POP1 is up-regulated in TNBC and may contribute to poor clinical prognosis.

### POP1 confers proliferation by advancing the cell cycle in TNBC

**Fig. 2.**
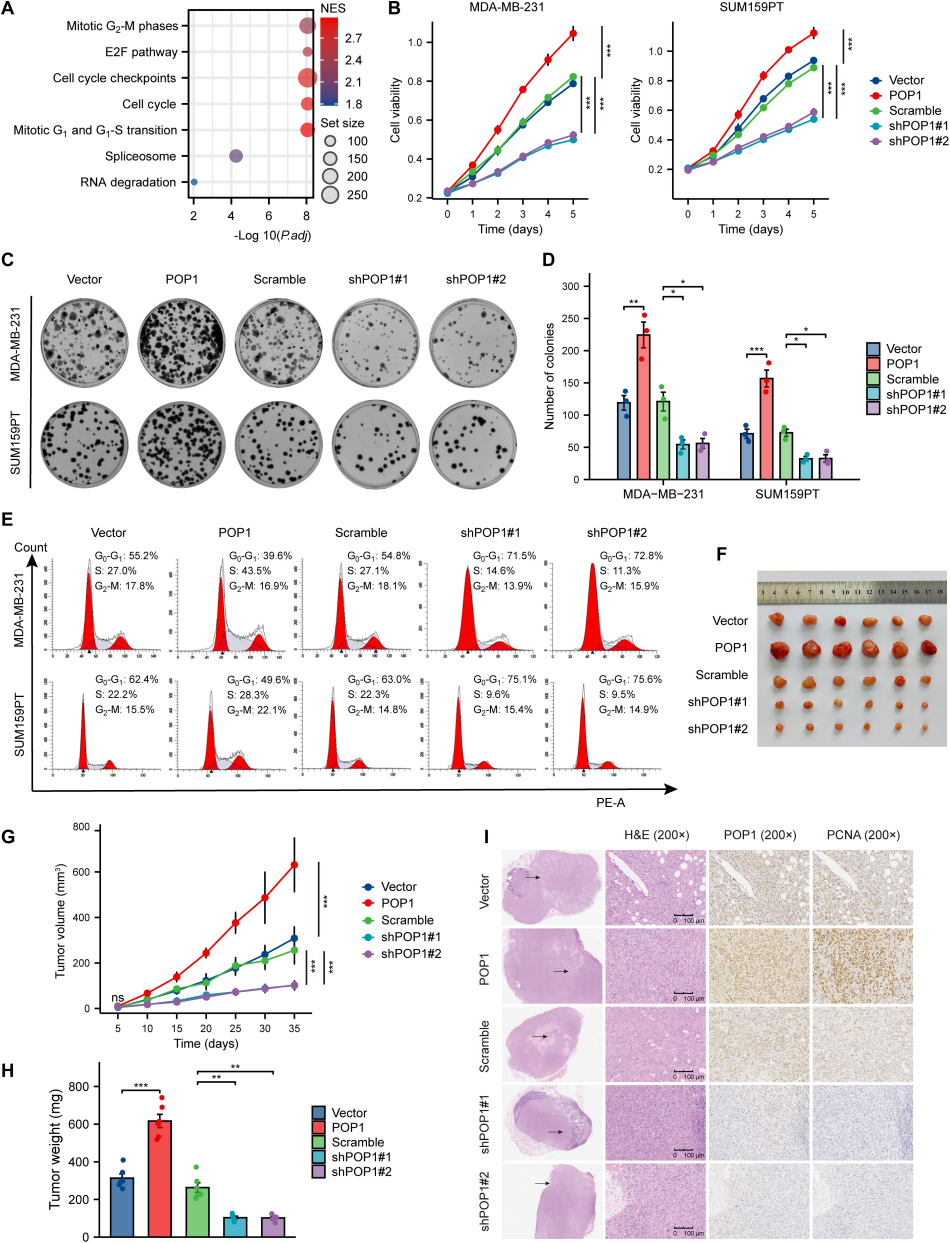
POP1 confers proliferation by advancing the cell cycle in TNBC. (A) Bubble diagram showing enrichment analysis results of POP1. (B) Line diagrams of CCK-8 proliferation experiment in TNBC cells with different POP1 expression levels. Statistical analyses were performed using the 2-way repeated-measures ANOVA. Typical results (C) and a statistical graph (D) of colony formation assay. One-way ANOVA was used for statistical analysis. (E) Representative flow cytometry cell cycle profiles. (F) Photos of subcutaneous tumors of different groups. (G) Tumor growth curves of different groups in subcutaneous tumor formation experiment in nude mice. Statistical analysis was performed using 2-way repeated-measures ANOVA. (H) Statistics of subcutaneous tumors’ weight in different groups. Welch one-way ANOVA test was used to analyze the differences among different groups. (I) Representative IHC images of subcutaneous tumors targeting POP1 and PCNA. *P < 0.05, **P < 0.01, ***P < 0.001.

To further clarify the role of POP1 in TNBC, we performed pathway enrichment analysis and gene set enrichment analysis (GSEA) analysis [24]. The results showed that POP1 was significantly enriched in proliferation-related biological processes including cell cycle, cell cycle checkpoint, and E2F pathway (Fig. 2A and Fig. S2A to C). We constructed stable POP1 overexpression and knockdown cell lines from 2 TNBC cells in which the expression of POP1 is moderate, MDA-MB-231 and SUM159PT, and verified both mRNA and protein expression of POP1 (Fig. S2D and E). Then, the Cell Counting Kit-8 (CCK-8) assay was used to assess the proliferation of cells with different POP1 expression levels. The results showed that high expression of POP1 significantly promoted the proliferation of TNBC cells, while knockdown POP1 had the opposite effect (Fig. 2B). Colony formation assay was used to compare the clonogenesis capacity of cells with different POP1 expression levels. Not surprisingly, TNBC cells with high POP1 expression had a stronger colony formation ability and vice versa (Fig. 2C and D).

Cell cycle is considered to be a key process affecting cell proliferation [25]. We then performed flow cytometry to compare the cell cycle distribution of TNBC cells with different POP1 expression levels. From the results, we found that the proportion of cells with high POP1 expression in the division quiescent phase (G_0_-G_1_) was lower than that in the control group, and the proportion in the mitotic phase (S and G_2_-M) was higher than that in the control group (Fig. 2E and Fig. S2F and G). To demonstrate functions of POP1 in vivo, we used the constructed stable MDA-MB-231 and then injected different groups of cells into the in situ fat pad of nude mouse mammary glands. The collected tumor photograph and tumor growth curves showed that overexpression of POP1 promoted the growth of TNBC cells, while knockdown of POP1 impaired the tumorigenic ability in vivo (Fig. 2F to H). The IHC results showed that POP1 expression was highly positively correlated with the proliferation molecular marker PCNA (Fig. 2I). Taken together, these results reveal that POP1 facilitates TNBC proliferation in vitro and in vivo.

### POP1 promotes decay of CDKN1A mRNA

**Fig. 3.**
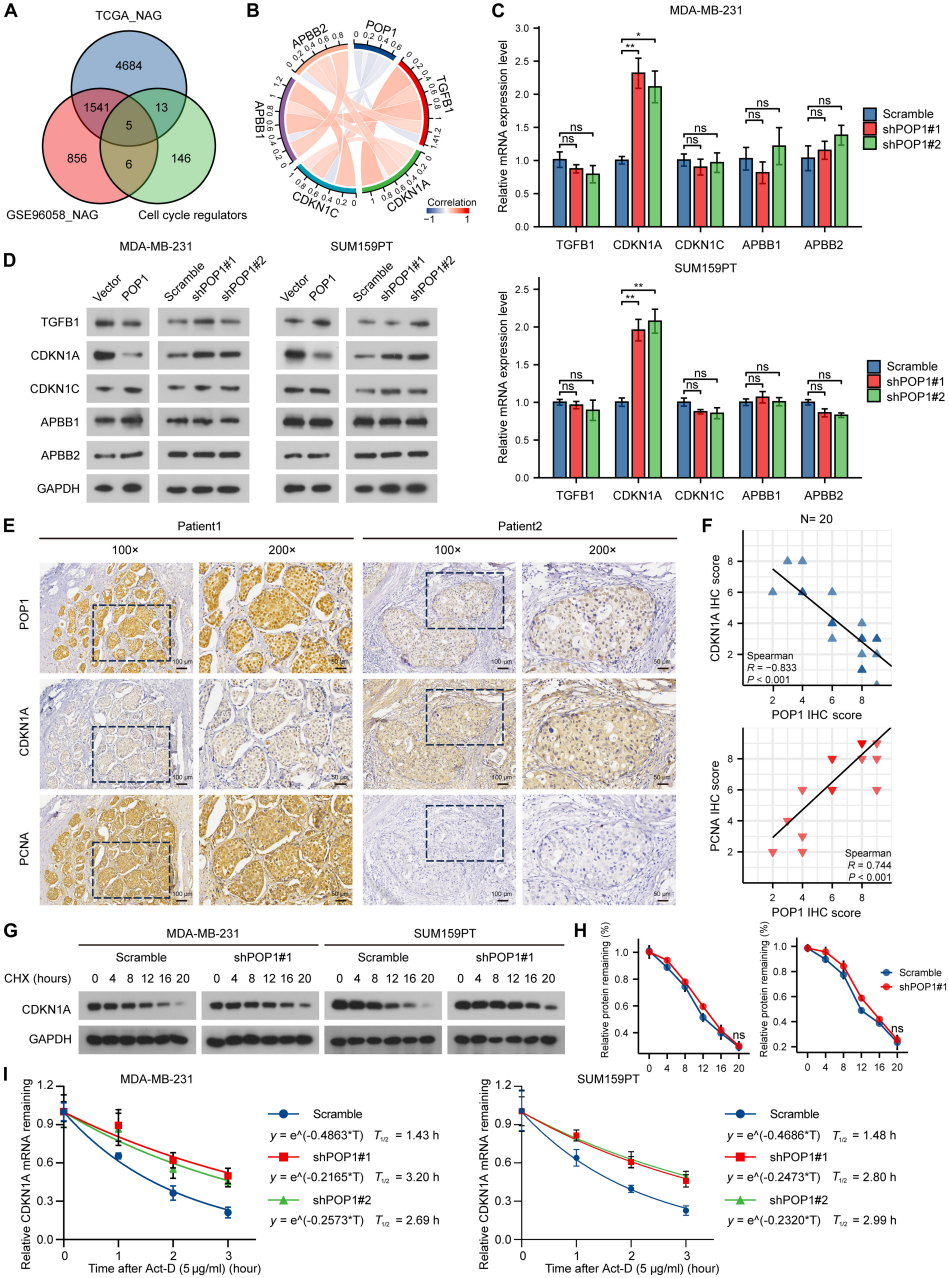
POP1 promotes decay of CDKN1A mRNA. (A) Venn diagram to screening out cell cycle regulators negatively associated with POP1 expression. (B) Chord diagram showing expression correlation between POP1 and APBB1, APBB2, CDKN1A, and CDKN1C and TGFB1. (C) mRNA expression of TGFB1, CDKN1A, CDKN1C, APBB1, and APBB2 in POP1 knockdown cells and control cells. (D) Protein levels of TGFB1, CDKN1A, CDKN1C, APBB1, and APBB2 in differentially expressed POP1 cells were explored by Western blot. (E) Representative IHC images of continuous sections showing the expression of POP1, CDKN1A, and PCNA in TNBC tissues. (F) Scatterplots of POP1, CDKN1A, and PCNA SI in TNBC tissues. Spearman correlation coefficient was used to evaluate the correlation. (G) Western blot analysis of CDKN1A degradation after treated with 100 mg/ml CHX. (H) Protein degradation curves of CDKN1A (based on the amount at 0 h after treatment). (I) Degradation curves and half-life of CDKN1A mRNA in MDA-MB-231 and SUM159PT after 5 mg/ml Act-D treatment. ns, not significant; *P < 0.05; **P < 0.01.

In order to clarify the mechanism of the RNase POP1 in regulating cell cycle and proliferation of TNBC, we first obtained negative-associated genes (NAGs) of POP1. Then, we screened out key molecules involved with cell cycle regulation through a Venn diagram (Fig. 3A). Express correlation chord chart suggested a negative correlation between expression of POP1 and APBB1, APBB2, CDKN1A, CDKN1C, and TGFB1 (Fig. 3B). We then examined mRNA and protein level of these 5 genes in POP1-overexpressed or knockdown cells, respectively. The results showed that only the expression of CDKN1A changed with the change of POP1 expression (Fig. 3C and D), suggesting that CDKN1A may be a downstream target of POP1. To verify the relationship between the 2, IHC staining was implemented on continuous tissue sections of 20 TNBC patients. The results showed that the SI of POP1 was negatively correlated with CDKN1A (Spearman *R* = −0.833, *P* < 0.01) and positively correlated with PCNA (Spearman *R* = 0.744, *P* < 0.01) (Fig. 3E and F). Furthermore, by referring to the pathology reports of these patients through the medical record system, we found that there is a significant positive correlation between POP1 expression in the tissue and the Ki-67 positivity rate (Spearman *R* = 0.572, *P* = 0.008) (Fig. S2H).

Based on these results, it is clear that POP1 down-regulates intracellular CDKN1A expression, but how this process is achieved remains unclear. After using 100 mg/ml cycloheximide (CHX) to inhibit intracellular protein synthesis, we detected CDKN1A protein level in TNBC cells at different time points. Results showed that CHX treatment did not delay the degradation time of CDKN1A protein in POP1 knockdown cells (Fig. 3G and H). Notably, when we applied 5 μg/ml actinomycin D (Act-D) to inhibit transcription, POP1 knockdown significantly prolonged the half-life of CDKN1A (1.43 h versus 3.20 h and 2.69 h; 1.48 h versus 2.80 h and 2.99 h) (Fig. 3I and J). Collectively, POP1 down-regulates the expression of CDKN1A at the mRNA level.

### POP1 degrades CDKN1A mRNA by interacting with its CDS

**Fig. 4.**
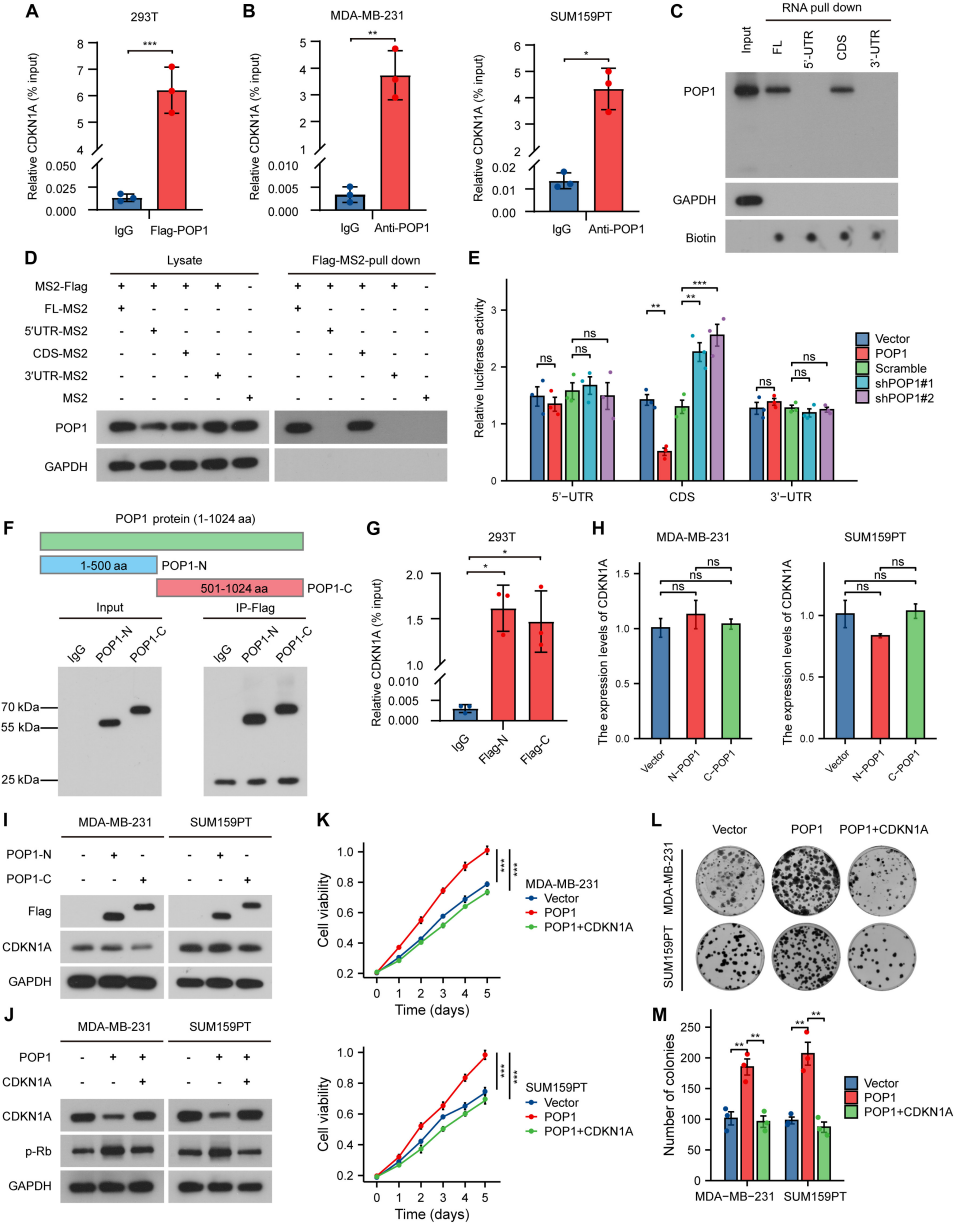
POP1 degrades CDKN1A mRNA by interacting with its CDS. RIP-PCR results about the binding between CDKN1A mRNA and POP1 protein and in 293T cells (A), MDA-MB-231 cells, and SUM159PT cells (B). (C) Western blot analysis of POP1 after biotin-labeled CDKN1A mRNA pull down. (D) Western blot analysis of POP1 after endogenous MS2 pull-down assay. (E) Luciferase reporter assay to explore the effect of POP1 on the decay of 5′UTR, CDS, and 3′UTR of CDKN1A. (F) Design and expression verification of POP1 truncated domain. (G) RIP-PCR assay to explore the binding between CDKN1A mRNA and the N-terminal or C-terminal truncate of POP1. (H) qPCR analysis to detect the effect of POP1 truncation on CDKN1A mRNA. (I) Western blot assay to detect the influence of POP1 truncation on CDKN1A protein. (J) Western blot analysis of p-Rb to detect the effect of CDKN1A overexpression on POP1 overexpression. (K) Cell viability curves showing the effect of rescue expression of CDKN1A on cell proliferation. Representative photographs (L) and a statistical graph (M) showing the effect of overexpression of CDKN1A on the colony formation ability of cells with high POP1 expression. *P < 0.05, **P < 0.01, ***P < 0.001.

Given that POP1 has RNase activity, we speculated whether POP1 down-regulates CDKN1A by degrading its mRNA directly. We performed exogenous and endogenous RNA immunoprecipitation (RIP) with anti-Flag antibody and anti-POP1 antibody, respectively, and detected CDKN1A mRNA by quantitative polymerase chain reaction (qPCR). The results showed that CDKN1A was enriched in the RNAs bound to POP1 (Fig. 4A and B). To detect the specific binding region, CDKN1A mRNA was divided into 3 different parts, including 5′ untranslated region (UTR), coding sequence (CDS), and 3′UTR, and transcribed in vitro with biotin labeling (Fig. S3A). RNA pull-down experiments were used to enrich proteins that bind to different pieces of RNA. Western blot images showed that POP1 bound to the CDS of CDKN1A rather than 5′UTR or 3′UTR (Fig. 4C). Based on the MS2 pull-down system, it was further confirmed that POP1 bound to the CDS region of CDKN1A mRNA (Fig. 4D and Fig. S3B). To explore the effect of the direct binding between POP1 and CDKN1A mRNA, we performed dual-luciferase reporter assay. We found that overexpression of POP1 down-regulates the expression of luciferase downstream of CDS region of CDKN1A and vice versa, which indicated that POP1 down-regulated the mRNA of CDKN1A by binding to its CDS region (Fig. 4E).

Considering that the current reports on POP1 protein domain are very limited, only the N-terminal domain (107 to 257 amino acids) and POPLD domain (617 to 708 amino acids) are known (Fig. S3C). We constructed truncated domains with the Flag tag containing the N terminus (1 to 500 amino acids) and C terminus (501 to 1,024 amino acids) of POP1 protein by querying the database. Western blot confirmed that labeled truncated POP1 was normally expressed and could be enriched by Flag magnetic beads (Fig. 4F). Further RIP-PCR experiment suggested that CDKN1A mRNA could bind to both N terminus and C terminus of POP1 protein (Fig. 4G). As for whether the N terminus or C terminus of POP1 played a key role in degrading CDKN1A mRNA, we introduced the truncated body separately in TNBC cells. Results of qPCR showed that single N terminus or C terminus did not affect intracellular CDKN1A mRNA levels (Fig. 4H). This suggests that although CDKN1A mRNA binds to both the N terminus and C terminus of POP1 protein, the truncated body does not have complete RNase activity. Not surprisingly, when we transfected the POP1 truncated body in the parent cells, the protein level of CDKN1A did not change significantly, either (Fig. 4I).

Above, we confirmed the direct regulatory effect of POP1 on CDKN1A mRNA. Then, we conducted simple rescue experiments to clarify the regulation of POP1 on the cell cycle through the degradation of CDKN1A. When we overexpressed CDKN1A in POP1-overexpressed TNBC cells, we noticed a significant reduction in the phosphorylation level of the cell cycle key checkpoint protein Rb (Fig. 4J and Fig. S3D). Moreover, overexpression of CDKN1A also impaired the proliferation ability and plate cloning ability of POP1-overexpressed cells (Fig. 4K to M). In summary, the results of this part showed that POP1 promoted TNBC proliferation by directly binding to the CDS region to degrade CDKN1A mRNA.

### POP1 degrades CDKN1A mRNA in an m6A-dependent manner

**Fig. 5.**
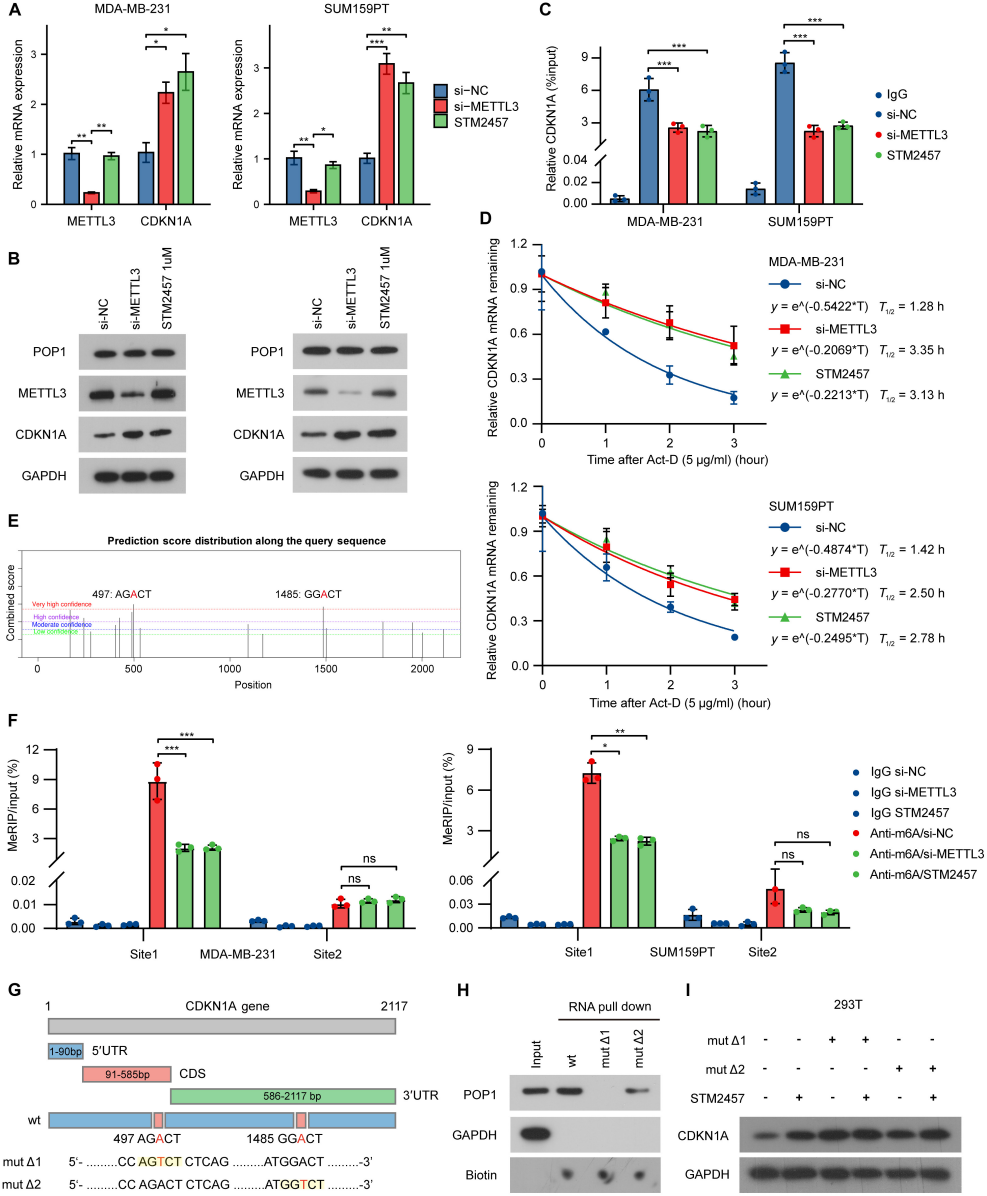
POP1 degrades CDKN1A mRNA in an m6A-dependent manner. qPCR and Western blot analysis of mRNA (A) and protein (B) of CDKN1A after inhibition of m6A modification by si-METTL3 or STM2457 treatment. (C) RIP-PCR used to detect the binding of POP1 and CDKN1A mRNA after inhibition of m6A modification. (D) Degradation curves and half-life of CDKN1A mRNA in MDA-MB-231 and SUM159PT after inhibition of m6A modification. (E) Potential m6A modification sites of CDKN1A mRNA predicted by SRAMP. (F) MeRIP-PCR used to detect m6A levels at 2 potential sites of CDKN1A after inhibiting m6A modification. (G) Schematic diagram of CDKN1A mutant at 2 sites. (H) RNA pull-down assay showing the binding of wild type and site mutant CDKN1A to POP1. (I) Western blot to detect the effect of STM2457 on mutant CDKN1A.

Previous studies have shown that POP1 could participate in the degradation of RNA with m6A modification. In this study, we have confirmed that POP1 has the function of degrading CDKN1A mRNA. We wondered whether the degradation process depended on the m6A modification of CDKN1A. STM2457 is a proven effective inhibitor of the m6A writer complex METTL3/METTL14 [26]. First, we explored the effect of STM2457 concentration on TNBC cells and on CDKN1A expression (Fig. S4A to E). We then examined the mRNA and protein expression of CDKN1A in TNBC cells treated with METTL3 knockdown and STM2457. As expected, both knockdown and inhibition of METTL3 could up-regulate CDKN1A expression (Fig. 5A and B). RIP-PCR experiments were repeated to determine whether knockdown METTL3 and STM2457 treatment up-regulated CDKN1A by inhibiting the interaction between POP1 and CDKN1A. The results showed that inhibition of m6A significantly reduced the binding of POP1 to CDKN1A (Fig. 5C). Further detection also suggested that inhibition of m6A by knocking down METTL3 or STM2457 treatment significantly prolonged the half-life of CDKN1A mRNA (Fig. 5D). These results indicated that degradation of CDKN1A mRNA by POP1 could be regulated via m6A modification.

Next, we attempted to explore the details of m6A modification of CDKN1A mRNA. By consulting the online SRAMP database (http://www.cuilab.cn/sramp/) [27], we predicted the potential m6A modification sites of CDKN1A mRNA. The results suggested that the 497th AGACT near the 3′ end of the CDS region and the 1485th GGACT in the 3′UTR region had the highest confidence among all potential sites (Fig. 5E). In order to identify the m6A modification site of CDKN1A, we performed methylated RIP (MeRIP)-PCR to detect the m6A modification at 2 sites in 2 TNBC cells. The results showed that knocking down METTL3 or STM2457 could significantly reduce the enrichment of sequences near the 497th AGACT but had little effect on the enrichment level of sequences near the 1485 AGACT (Fig. 5F). To further verify the effect of m6A modification at specific sites of CDKN1A mRNA, we constructed CDKN1A with mutations in these 2 potential sites, namely, mutΔ1 and mutΔ2 (Fig. 5G). RNA pull-down assay showed that CDKN1A mRNA of wild-type and mutΔ2 could bind to POP1 while mutΔ1 no longer bound to POP1 (Fig. 5H). These results suggested that the interaction of POP1 with CDKN1A mRNA was dependent on m6A modification at position 497 of the mRNA. In addition, Western blot analysis of CDKN1A showed that STM2457 could not up-regulate the expression of mutΔ1 compared with the wild type and mutΔ2 (Fig. 5I and Fig. S4F). Taken together, POP1 degrades CDKN1A mRNA depending on its m6A modification at site 497.

### YTHDF2 is the m6A reader mediating CDKN1A degradation

**Fig. 6.**
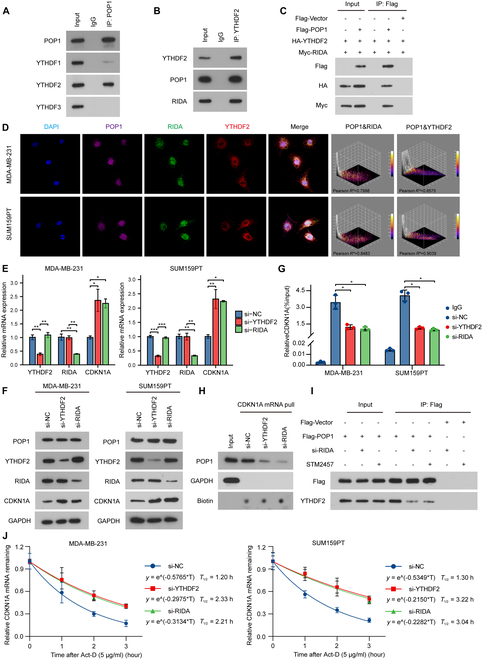
YTHDF2 is the m6A reader mediating CDKN1A degradation. (A) Co-IP used to detect the binding of POP1 with YTH m6A RNA binding proteins. (B) Co-IP to detect the binding of YTHDF2 with POP1 and RIDA. (C) Exogenous co-IP to detect the binding of POP1, YTHDF2, and RIDA. (D) Immunofluorescence images showing subcellular localization and colocalization of POP1, RIDA, and YTHDF2. qPCR and Western blot analysis of mRNA (E) and protein (F) of CDKN1A after inhibition of m6A reading by si-YTHDF2 or si-RIDA. RIP-PCR (G) and RNA pull down (H) used to detect the binding of POP1 and CDKN1A mRNA after inhibition of m6A recognition. Welch one-way ANOVA test was used for difference analysis according to the data’s normality and homogeneity of variance. (I) Co-IP to detect the effect on the binding of POP1 with YTHDF2 when knocking down RIDA or treating with STM2457. (J) Degradation curves and half-life of CDKN1A mRNA in MDA-MB-231 and SUM159PT after inhibiting m6A recognition.

After it was confirmed that POP1 degraded CDKN1A mRNA in an m6A-dependent manner, the reader mediating this process became a problem that needed to be clarified. We first performed co-IP to validate widely studied YTH m6A RNA binding proteins including YTHDF1, YTHDF2, and YTHDF3. Among the proteins bound to POP1, YTHDF2 was significantly enriched (Fig. 6A). Among the proteins bound to YTHDF2, POP1 and a known adaptor protein, RIDA, were enriched (Fig. 6B). Not only that, we repeated the co-IP of the exogenous protein. By transfecting labeled POP1, YTHDF2, and RIDA, the corresponding label content was detected after enrichment. The results showed that POP1 combined directly with YTHDF2 and RIDA (Fig. 6C). High-power (60×) images of immunofluorescence show the location characteristics of these 3 molecules within the cell. Further analysis by ImageJ indicated that POP1, RIDA, and YTHDF2 had a high degree of adhesion (most Pearson *R*^2^ ≥ 0.8) (Fig. 6D). These results suggested that YTHDF2 bound to POP1, so we speculated that YTHDF2 was the reader mediating CDKN1A degradation.

Subsequently, qPCR and Western blot results showed that knocking down YTHDF2 and RIDA could significantly up-regulate the mRNA and protein levels of CDKN1A without influencing its m6A modification in TNBC cells (Fig. 6E and F and Fig. S4G). These suggested that they may play an important role in mediating CDKN1A mRNA degradation. Further RIP-PCR and RNA pull-down experiments also showed that knocking down either YTHDF2 or RIDA significantly impaired the interaction between POP1 and CDKN1A mRNA (Fig. 6G and H). It was worth noting that whether knockdown of the ligation helper protein RIDA or using STM2457 inhibited m6A modification, the binding of POP1 with YTHDF2 was significantly reduced (Fig. 6I). Finally, knocking down YTHDF2 or RIDA to inhibit m6A recognition significantly extended the half-life of CDKN1A mRNA (Fig. 6J), just as inhibiting m6A modification. Collectively, we have demonstrated that YTHDF2 was the key reader mediating the degradation of CDKN1A mRNA in TNBC, which was consistent with the reported role of YTHDF2 in RNA decay in previous studies.

### Promotion of TNBC proliferation by POP1 depends on m6A modification and recognition of CDKN1A

**Fig. 7.**
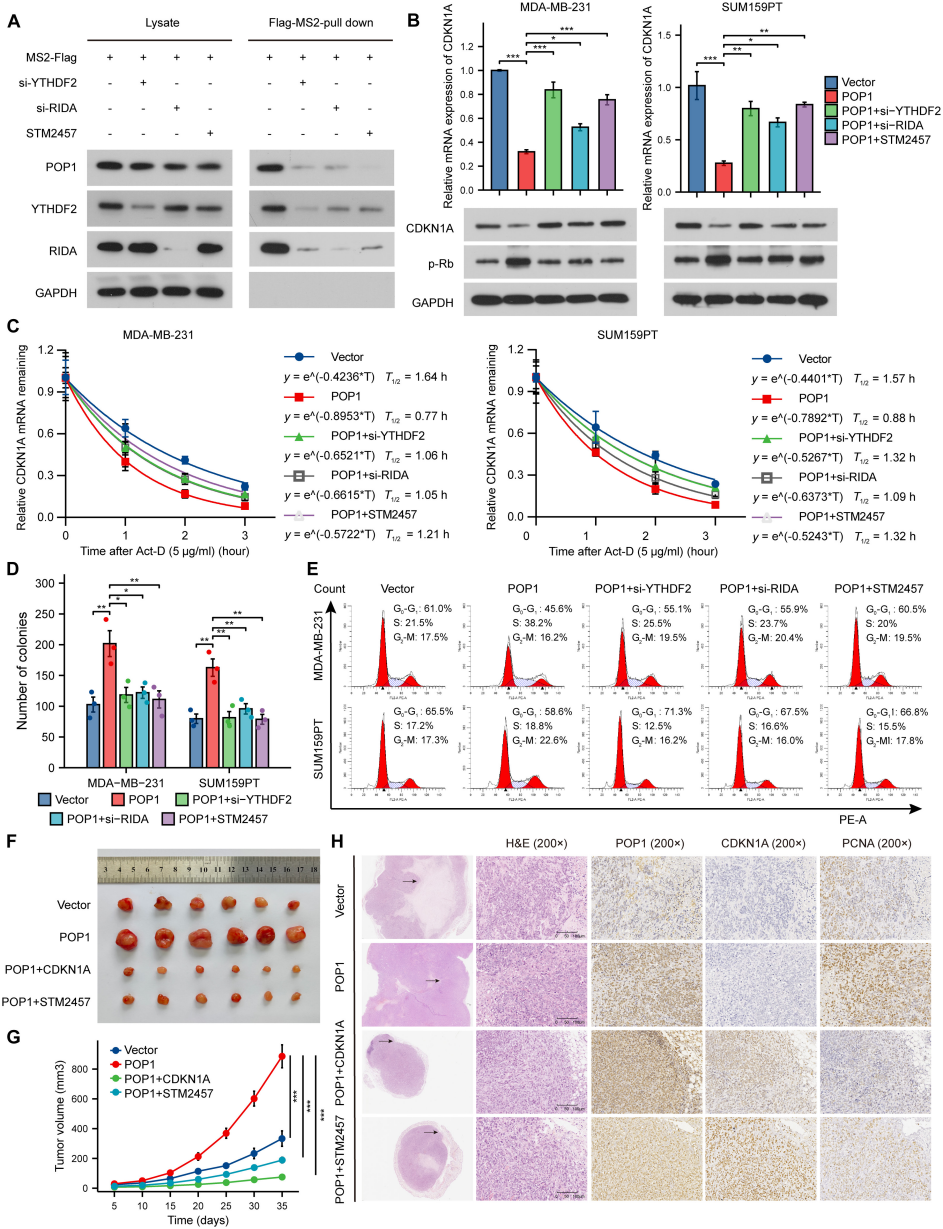
Promotion of TNBC proliferation by POP1 depends on m6A modification and recognition of CDKN1A. (A) The MS2 pull-down experiment demonstrated the combination of POP1, YTHDF2, and RIDA with CDKN1A mRNA under si-YTHDF2, si-RIDA, and STM2457 treatment. (B) qPCR and Western blot analyses of the effects of interference with m6A modification and recognition on CDKN1A expression and Rb phosphorylation in POP1-overexpressed cells. (C) Degradation curves and half-life of CDKN1A mRNA in POP1-overexpressed cells after inhibiting m6A modification or recognition. (D) Statistical results of colony formation assay of POP1-overexpressed TNBC cells under different treatments. (E) Representative flow cytometry cell cycle profiles of POP1-overexpressed TNBC cells under different treatments. Subcutaneous tumor photos (F) and tumor volume growth curves (G) of TNBC cells in different treatment groups. Statistical analysis of tumor volume growth in different groups was conducted using 2-way repeated-measures ANOVA. (H) Representative IHC images of subcutaneous tumorstargeting POP1, CDKN1A, and PCNA. *P < 0.05, **P < 0.01, ***P < 0.001.

The effect of inhibiting m6A modification and recognition process on the binding of CDKN1A mRNA to POP1, YTHDF2, and RIDA was detected by MS2-based RNA pull down. The results suggested the necessity of both m6A modification and recognition in this regulatory process (Fig. 7A). So far, we have basically demonstrated that YTHDF2-RIDA-POP1 were key elements in degrading CDKN1A mRNA in an m6A-dependent manner. In order to determine whether the promotion of TNBC proliferation by POP1 depends on this function, we conducted necessary rescue experiments. First, by interfering with the m6A modification and recognition process with si-YTHDF2, si-RIDA, or STM2457 treatment, the up-regulation of CDKN1A in POP1-overexpressing cells was restored and the cell proliferation marker p-Rb was significantly reduced (Fig. 7B). Subsequent RNA half-life measurements showed clearly that both m6A modification and recognition were important for the reduction of CDKN1A half-life in POP1-overexpressing cells (Fig. 7C). Consistent with the expression of p-Rb in TNBC cells, inhibiting m6A modification or recognition of cells could also significantly reduce the colony formation ability and the proportion of proliferating cells in cells with high POP1 expression (Fig. 7D and E and Fig. S5A to C). The results of tumor formation experiment in nude mice showed that overexpression of CDKN1A or treatment with STM2457 in cells with high POP1 expression greatly impaired the tumor formation ability (Fig. 7F). It is obvious that restoring intracellular CDKN1A expression by different methods could block POP1-mediated high proliferation (Fig. 7G and Fig. S5D). These results were again confirmed by IHC staining of POP1, CDKN1A, and proliferating marker PCNA in successive subcutaneous tumor sections (Fig. 7H). In short, the expression of CDKN1A was a key indicator of proliferation, and POP1 promoted TNBC cell proliferation by down-regulating CDKN1A in an m6A-dependent manner.

### Clinical significance of POP1 and potential application of STM2457 in TNBC

**Fig. 8.**
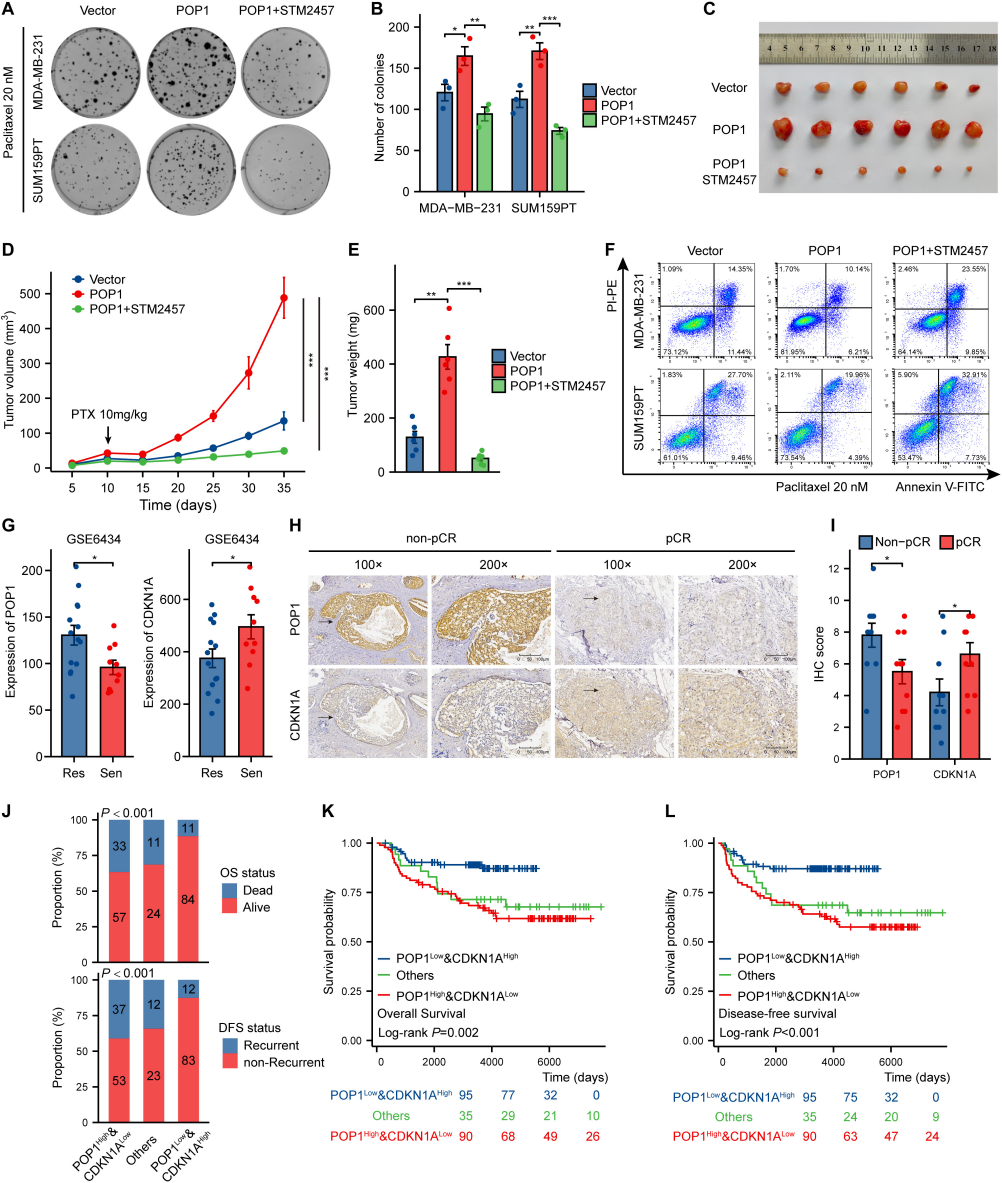
Clinical significance of POP1 and potential application of STM2457 in TNBC. (A) Colony formation pictures to show the effect of m6A inhibitor STM2457 on the response of TNBC cells to paclitaxel. (B) Statistical graph of the results of the colony formation assay. Subcutaneous tumor photos (C), tumor growth curves (D), and statistics of tumor weight (E) of TNBC cells showing the effect of STM2457 on sensitivity to paclitaxel in vivo. (F) Representative Annexin V-PI double dye flow cytometry results showing the effect of STM2457 on apoptosis of TNBC cells treated with 20 nM paclitaxel. (G) Expression of POP1 and CDKN1A mRNA in tissues of patients with paclitaxel sensitivity and resistance in the dataset GSE6434. (H) Representative IHC images of continuous sections showing the expression of POP1 and CDKN1A in patients with different response to neoadjuvant chemotherapy. (I) POP1 and CDKN1A expression in non-pathological complete response (pCR) patients compared with pCR patients (non-pCR *N* = 10, pCR *N* = 10). (J) Proportion of POP1 and CDKN1A expression in different OS and DFS outcomes. The statistics was conducted using the chi-square test. Survival curves of patients with different levels of POP1 and CDKN1A expression including OS (K) and DFS (L). Log-rank test was used for survival analysis of different groups. *P < 0.05, **P < 0.01, ***P < 0.001.

Previous studies have reported that down-regulation of CDKN1A is not only related to the uncontrolled proliferation of cancer cells but also responsible for the resistance of some cell cycle-specific drugs such as CDK4/6 inhibitors and paclitaxel [28–30]. Here, we explored the influence of POP1 and CDKN1A expression on paclitaxel sensitivity and tried to find predictors of TNBC chemotherapy sensitivity and potential solutions for chemotherapy resistance. First, the colony formation experiment showed that POP1 overexpression could enhance the colony formation ability of TNBC cells treated with 20 μM paclitaxel, and STM2457 combined with it significantly enhanced the sensitivity of TNBC cells to paclitaxel (Fig. 8A and B). In vivo experiments show similar results. Treatment with 10 mg/kg of paclitaxel at 10 d after tumor formation and treatment with 50 mg/kg of STM2457 significantly improved paclitaxel sensitivity, which was reflected in significant differences in tumor growth curve, tumor size, and tumor weight (Fig. 8C to E). Annexin V-propidium iodide (PI) flow cytometry also showed that STM2457 distinctly reduced the survival rate of the cells under the treatment of paclitaxel (Fig. 8F and Fig. S6A). So far, our results have indicated that intervention of CDKN1A degradation by STM2457 had a promising role in the sensitizing effect of paclitaxel.

**Fig. 9.**
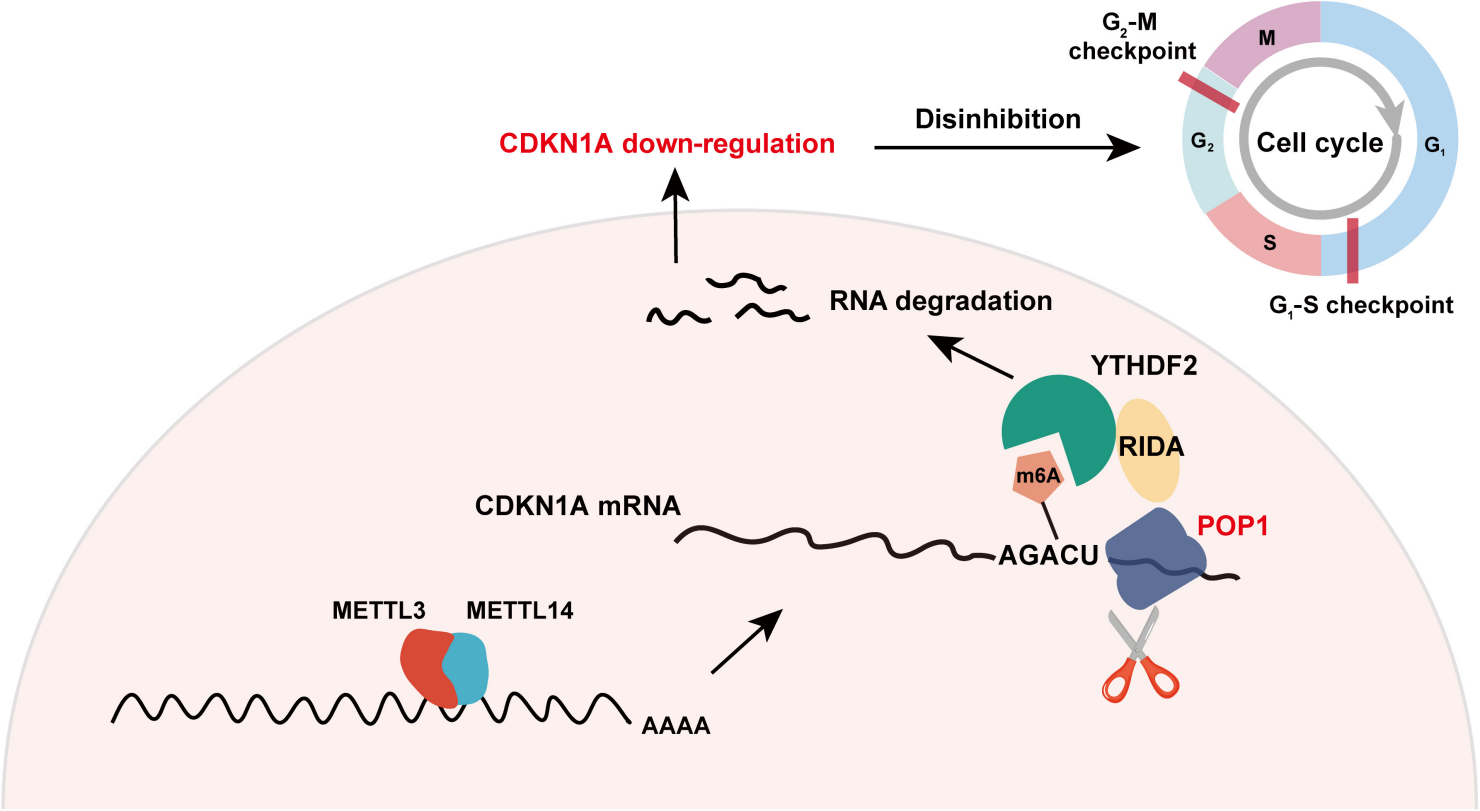
A model of this study showing that the YTHDF2–RIDA–POP1–CDKN1A axis promotes proliferation in TNBC.

As to whether the expression of POP1 and CDKN1A played a certain role in predicting TNBC chemotherapy sensitivity and patient prognosis, we first referred to the online dataset GSE6434 [31]. It was clear that POP1 expression was higher and CDKN1A expression was lower in the tissues of 14 paclitaxel-resistant patients compared with 10 sensitive patients (Fig. 8G). IHC staining of tissue sections of patients receiving neoadjuvant chemotherapy based on paclitaxel in our hospital also showed similar results (Fig. 8H and I). We did IHC staining on the tissues of 220 patients in our cohort and divided them into 3 groups, namely, POP1^High^&CDKN1A^Low^, POP1^Low^&CDKN1A^High^, and Others, for survival analysis and comparison. The results showed that patients with high POP1 expression and low CDKN1A expression had the worst prognosis (Fig. 8J to L), and POP1 combined with CDKN1A predicted the prognosis more effectively than POP1 alone (Fig. S6B to G). Taken together, POP1 regulating CDKN1A degradation provided potential markers for prediction of TNBC chemotherapy response and prognosis and a potential strategy for sensitization chemotherapy. Finally, a model diagram summarized the content of this study (Fig. 9).

## Discussion

Uncontrolled proliferation is a common feature of all malignant tumors, and it is also one of the most important causes of organ damage and patient death. Among the 4 common molecular subtypes of breast cancer, HER2 overexpression type and TNBC have significantly greater proliferation capacity than luminal type [32,33]. This is often reflected in the IHC results of patient tissue sections; the positive rate of Ki-67, a key indicator of proliferation, is often more than 50%; and some patients can even reach 80% to 90%. A higher rate of proliferation often indicates a more dangerous condition. This group of patients often face a very rapid rate of tumor progression and early distant metastasis, which are closely associated with poor prognosis. At present, the regulatory mechanism of hyperproliferation of HER2-overexpressed breast cancer has been clarified [34]. Fortunately, the monoclonal antibodies against HER2, trastuzumab and pertuzumab, as well as the small-molecule inhibitor lapatinib, have been clinically proven to have surprising efficacy and greatly improve the prognosis of patients [35–37]. However, the key genes and mechanisms driving the proliferation of TNBC remain inadequately understood, contributing to the limited availability of effective therapeutic targets and strategies beyond chemotherapy.. Therefore, in-depth research on the regulatory mechanisms and critical genes regulating cell cycle and hyperproliferation in TNBC is crucial for improving the treatment status.

It is well known that the cell cycle is regulated by a network of genes. When cells receive external signals to initiate proliferation, expression of intracellular cyclins is up-regulated, which phosphorylates the key checkpoint gene Rb and releases E2F by activating cycle-dependent kinases (CDKs) [38,39], ultimately causing cell proliferation. Correspondingly, a class of molecules called cyclin-dependent kinase inhibitor (CKI), which negatively regulates the cell cycle, also exists in the cell. It contains Ink4, which inhibits only CDK4/6, and kinase inhibition protein (Kip), which inhibits multiple CDKs simultaneously [40,41]. CDKN1A, also known as p21, is one of the most important Kip. As the downstream gene of the tumor suppressor gene p53, it has the function of potently inhibiting the cell cycle through various pathways. This includes but is not limited to inhibiting the activity of most CDKs, and binding to PCNA, a key cofactor in DNA synthesis, and directly inhibits DNA synthesis [38,42]. CDKN1A has been shown to be down-regulated in multiple malignant tumors, with its reduced expression or inactivating mutations closely linked to tumor progression. The functional status of p53-CDKN1A is also related to the efficacy of multiple chemotherapy agents and radiotherapy [43]. When CDKN1A is inactivated or down-regulated, apoptosis or senescence of cells is inhibited, resulting in drug resistance. Moreover, in breast cancer, the loss of CDKN1A expression greatly reduces the sensitivity of cell cycle-related drugs such as CDK4/6 inhibitors and paclitaxel [42]. It is worth noting that inducing CDKN1A expression or stability by a variety of methods has shown remarkable effects in inhibiting tumor progression and enhancing the therapeutic sensitivity of tumors.

In this study, we found that POP1 is specifically highly expressed in TNBC tissues and is associated with poor prognosis, which is consistent with the conclusion of a recent study [44]. However, there are limited reports concerning the function of POP1 in mammals. A recent study showed that similar to its function in yeast, POP1 also promotes tumor progression in human tumors by keeping telomeres intact [44]. As an RBP component shared by RNase P/MRP, POP1 was reported to be involved in the posttranscriptional processing and maturation of rRNA and tRNA in previous studies. The study by Park et al. [20] offers new insights into the function of RNase P/MRP, namely, one of the readers of m6A, YTHDF2, mediates the decay of some m6A-modified RNAs via RNase P/MRP in a RIDA-dependent manner. This coincides with the enrichment analysis in this study. These evidences allow us to focus on molecules that are negatively regulated by POP1 and closely related to the cell cycle, following the confirmation of POP1’s role in promoting TNBC cell cycle and proliferation. Through a sequence of experiments, we identified and validated that CDKN1A mRNA as a substrate for POP1 degrades in an m6A-dependent manner. Moreover, we confirmed that YTHDF2, rather than other readers, plays a key recognition role in this process, which is also consistent with the previous studies that YTHDF2 often mediates the down-regulation of m6A-modified RNA. However, the details of degradation of m6A substrates based on the YTHDF2/RIDA/POP1 axis are still worthy of further exploration and research. This regulatory axis was first reported roughly by Yoon et al., and the results in this study further confirm that YTHDF2-mediated down-regulation of m6A-modified mRNA is partially dependent on RIDA [20]. Considering that the RNase P/MRP where POP1 is located is a relatively large protein complex, how YTHDF2 is guided by RIDA to bind to POP1 after recognizing m6A, and how the RNA substrate overcomes steric hindrance and fully degrades the enzyme in the case of binding numerous protein molecules, is still worthy of further investigation.

When it comes to m6A, tremendous research has been reported in recent 3 years. The dysregulation or dysfunction of m6A writers, erasers, and readers can lead to changes in the m6A modification status of RNA or changes in the downstream effects of the modification, and eventually lead to abnormal expression of related genes [11]. This is involved in the regulation of almost all hallmarks of cancer, along with influencing the efficacy of many current interventions [12,45]. Therefore, many small-molecule compounds involved in the regulation of m6A have been developed to improve the treatment status of some diseases. STM2457, a compound that reduces m6A modification by suppressing the METTL3/METTL14 complex, has shown good antitumor effects in malignant tumors including leukemia, cholangiocarcinoma, and osteosarcoma and has shown potential in reversing liver and lung cancer resistance [26,46–48]. In this study, our results also suggested that STM2457 significantly inhibits CDKN1A proliferation and increases the sensitivity to paclitaxel by restoring expression of CDKN1A in TNBC. This suggests the potential of STM2457 in improving the prognosis of TNBC patients in the future.

This study has some potential limitations. First, when we were looking for the downstream POP1, it was only realized through the analysis of public databases, and it was not verified by the corresponding sequencing means. According to previous studies, in addition to RNase P/MRP, the CCR4-NOT complex can also participate in YTHDF2-mediated RNA degradation. In this study, we could not prove that POP1 is the only component of CDKN1A-dependent m6A degradation. In addition, in the process of searching for the reader mediating CDKN1A degradation, we did not verify all readers after confirming the role of YTHDF2. Finally, due to the unavailability of commercial antibodies for IHC detection of mouse POP1, we had to abandon the constructed 4T1 cell line and use human MDA-MB-231 in vivo experiments.

In conclusion, our study revealed that POP1 is up-regulated in TNBC and promotes hyperproliferation of TNBC by degrading CDKN1A in an m6A-dependent manner. Our findings suggest the important role of POP1 in regulating TNBC proliferation and provide new prognostic markers and potential therapeutic strategies for TNBC.

## Materials and Methods

### Patient information and tissue specimens

Tumor tissues were from surgical excision samples of patients pathologically diagnosed in Sun Yat-sen University Cancer Center (SYSUCC) from 2001 to 2012, and were obtained from the specimen bank of SYSUCC. The frozen tissues were used for qPCR and Western blot experiments, and the paraffin-embedded samples were used for IHC. Clinical information and follow-up information were obtained from the hospital’s medical record system. We kept all personal data confidential and conducted this study in accordance with the Declaration of Helsinki. All patients signed informed consent, and this study was approved by the Ethics Committee of SYSUCC to use these clinically relevant materials.

### Cell lines and cell culture

All cells including the breast epithelial cell line MCF10A, breast cancer cell lines, and 293T were obtained from the State Key Laboratory of Oncology in South China (SYSUCC, Guangzhou, China). All cell lines were cultured in the American Type Culture Collection-recommended medium (Gibco) with 10% fetal bovine serum at 37 °C and 5% CO_2_. Before carrying out experiments, cells were examined for mycoplasma contamination by short tandem repeat analysis using the LookOut Mycoplasma PCR Detection Kit (#MP0035, Sigma-Aldrich). All cells used in the experiments were within 5 generations from thawing of cryopreserved cells.

### Establishment of stable cell lines and siRNA transfection

All stable cell lines were generated from MDA-MB-231 and SUM159PT infected with retrovirus. The open reading frame sequences of POP1 and CDKN1A inserted into the pLVX-hygro vector for overexpression and short hairpin RNA (shRNA) targeting POP1 were cloned into the pSuper-retro-hygro vector. The sequence of shRNA was provided in Table S1. Target plasmid and package plasmid were cotransfected into 293T. The virus in the medium was collected and concentrated, and then the parent cells were infected for 3 d. After screening for 250 μg/ml hygromycin for 3 d, cells were amplified. The efficiency of overexpression and knockdown was verified by qPCR and Western blot.

The synthesized small interfering RNAs (siRNAs) were used at 20 nM with Lipofectamine 2000 reagents (#11668500, Invitrogen) according to user instructions. Knockdown efficiency of mRNA or protein was collected 48 h after transfection and analyzed by qPCR and Western blot. The sequence of siRNA was described in Table S1.

### RNA extraction, reverse transcription, and qPCR

RNA from different cells and tissues was extracted using the TRIzol reagent (#15596018CN, Invitrogen) according to the instructive protocol. The extracted RNA was treated with RNase-free deoxyribonuclease (DNase) and reverse-transcribed according to the instructions of the kit, and cDNA was obtained (#A0010CGQ, EZBioscience). Subsequent qPCR was performed in strict accordance with the user instructions, and gene expression level was normalized to glyceraldehyde-3-phosphate dehydrogenase (GAPDH) (#CFX96, Bio-Rad; #A0001-R1, EZBioscience). All primers used in this study were summarized in Table S1.

### Western blot

The protein was extracted according to the standard experimental protocol, denatured, and analyzed by Western blot. Protein expression level was normalized to GAPDH. All the antibodies used in this study including primary and secondary antibodies were summarized in Table S2.

### Cell viability assay

Different groups of cells were implanted on 96-well plates for 48 h. CCK-8 is used to detect cell proliferation according to the instructions for use (#C0037, Beyotime). After adding CCK-8 reagent, the culture was kept away from light at 37 °C for 2 h. The absorbance of each group at 450 nm was then measured using a microplate reader (#EPOCH2, BioTek).

### Immunohistochemistry

We performed IHC of paraffin-embedded human breast cancer tissues and mouse mammary fat pad subcutaneous tumors. Labeled molecules, corresponding antibodies, and dilution ratios were described in Table S2. This experiment was completed with the kit in strict accordance with the steps of the instructions (#PV6000, ZSGB-BIO). IHC staining was independently scored by 2 uninformed pathologists. Tumor cell proportions were scored as follows: 0: no positive cells, 1: <10%, 2: 10% to 35%, 3: 35% to 75%, 4: >75%. Staining intensity was graded as follows: 1: no staining, 2: weak staining (light yellow), 3: moderate staining (yellow brown), 4: strong staining (brown). The staining index (SI) was calculated by multiplying the proportion of positive cells and the staining intensity. High and low expression of POP1, CDKN1A, and proliferating cell nuclear antigen (PCNA) were defined as SI ≥ 6 or SI < 6, and log-rank test was performed accordingly to statistically compare OS and disease-free survival (DFS) of these 220 patients. All antibodies used and their dilution ratios were summarized in Table S2.

### Immunofluorescence

Cells were immobilized with 4% paraformaldehyde for 30 min after phosphate-buffered saline (PBS) cleaning for 3 times. They were then treated with PBST containing 0.3%Triton X-100 and 1% bovine serum albumin (BSA) at room temperature for 30 min. The primary antibody was incubated at 4 °C overnight. The labeled cells were cleaned 3 times and incubated with fluorescent secondary antibody corresponding to the reactivity at room temperature for 1 h, and then the process of sealing and antibody incubation was repeated until POP1, YTHDF2, and RIDA were all labeled. 4′,6-Diamidino-2-phenylindole (DAPI) was used to dye the nucleus for 10 min (#HY-D0814, MedChemExpress). Cells were observed and photographed using a laser confocal microscope with 60× objective lens (#CSU-W1, Nikon). Colocalization analysis was performed using ImageJ. All antibodies used and their dilution ratios were provided in Table S2.

### Flow cytometry

The cell cycle was measured using a commercially available kit (#C1052, Beyotime) and in strict accordance with instructions. The cells of different groups were grown under the same culture conditions to a logarithmic stage after digestion, fixation, washing, and staining, and then were analyzed in the 488-nm channel (#CytoFLEX, BECKMAN). The cell debris removal method was applied to all groups, and the number of eligible particles in each group was ensured to exceed 20,000. After the experiment was completed, ModFit LT software (version 4.1.7) was used for further analysis. Apoptosis of cells under different treatment conditions was detected using another kit (#A211-02, Vazyme). The cells were digested and collected 48 h after paclitaxel treatment. The experiment was completed according to the protocol of the kit, and the results were analyzed and visualized using the CytoExpert software (version 2.4.0.28).

### Colony formation assay

TNBC cells in the logarithmic growth phase were trypsinized, counted, and then reseeded into 6-well plates at a density of 800 cells per well. The medium was replaced every 3 to 5 d until day 14. After fixation with 4% paraformaldehyde, the cells were stained with Giemsa for 20 min, washed, air-dried, photographed, and counted under a low-power microscope (4×). In this study, colonies containing fewer than 50 cells were excluded from the count.

### Luciferase reporter assay

We performed 5′UTR, CDS, and 3′UTR luciferase reporter assays of CDKN1A mRNA to assess whether POP1 induces CDKN1A mRNA decay through its binding to the CDS region. To put it simply, 20,000 cells were inoculated in a 48-well plate in triplicate and maintained for 24 h. The reporter plasmid (100 ng) along with 1 ng of pRL-TK Renilla plasmid (#E224A, Promega) was transfected into targeted cells using Lipofectamine 3000 reagent. According to the protocol provided by the manufacturer, a dual-luciferase reporting assay kit (#E1910, Promega) was applied to detect luciferase and Renilla signals 1 d after transfection. The relative activity of luciferase is calculated as the ratio of luciferase to Renilla signal.

### Protein immunoprecipitation assay

After the cell samples in the medium were collected and washed with PBS, the lysis buffer (150 mM NaCl, 10 mM HEPES, 1% NP-40, pH 7.4) was used to prepare the cell lysis solution on ice. The lysates were incubated with anti-POP1 antibody (#12029-1-AP, Proteintech) and protein G-conjugated magnetic beads (#HY-K0202, MedChemExpress) or anti-Flag magnetic beads (#HY-K0207, MedChemExpress) at 4 °C overnight. The rubber beads containing the affinity antibody binding protein were then washed with washing buffer (150 mM NaCl, 10 mM HEPES, 0.1% NP-40, pH 7.4) for 6 times and then eluted with 1 M glycine solution (pH 3.0). The eluent was detected by Western blot.

### RNA immunoprecipitation assay

RIP was used to detect direct interactions between CDKN1A mRNA and POP1 protein. Cells (1 × 10^7^) were digested, collected, and then fully decomposed in lysate (20 nM tris–HCl, pH 8.0, 10 mM NaCl, 1 nM EDTA, 0.5% NP-40) with 1× protease inhibitor (#G6521, Promega) and RNasin (#N2111, Promega). Cell lysis (50 μl) was cryopreserved as input. Other lysis was then incubated with anti-POP1 antibody (#12029-1-AP, Proteintech) and protein G-conjugated magnetic beads (#HY-K0202, MedChemExpress) or anti-Flag magnetic beads (#HY-K0207, MedChemExpress) at 4 °C overnight. After washing for 5 times with precooled RIP wash buffer (1× PBS, 0.1% SDS, 0.5% Nonidet P40), the Elution Buffer [5 mM tris–HCl, pH 7.5, 1 mM EDTA, pH 8.0, 0.05% SDS, 20 mg/ml proteinase K (#HY-108717, MedChemExpress)] was used to eluate the coprecipitated RNAs. RNA was extracted by TRIzol Reagent (#15596018CN, Invitrogen) and analyzed by qPCR. All primers used in this study were summarized in Table S1.

### RNA synthesis and pull down

Full-length and truncated CDKN1A were transcribed in vitro with T7 or SP6 RNA polymerase from templates amplified with T7 forward 5′-TAATACGACTCACTATAG-3′ and SP6 reverse 5′-ATTTAGGTGACACTATAG-3′ primers (#D2314, Beyotime). The obtained RNA fragments were then purified and labeled with biotin according to the Biotin RNA Labeling kit instructions (#R7061M, Beyotime). Then, the RNA pull-down experiment was performed. To put it simply, 1 × 10^7^ cells were lysed with 1 ml of buffer [50 nM tris–HCl, pH 7.9, 10% glycerol, 100 mM KCl, 5 mM MgCl_2_, 10 nM β-mercaptoethanol, 0.1% NP-40, 1 mM phenylmethylsulfonyl fluoride (#ST505, Beyotime), 1× Superase-in (#AM2694, Invitrogen), 1× protase inhibitor (#G6521, Promega), 10 mM KCl]. Labeled RNA (100 pmol) was incubated with cell lysate at room temperature for 1 h. Then, each group was incubated with 100 μl of cleaned streptavidin magnetic beads (#HY-K0208, Beyotime) and the reaction products above for 1 h. The treated beads were washed 4 to 5 times, and then the eluent was used to obtain the protein and subject to Western blot analysis.

### Methylated RNA immunoprecipitation

Total RNA from different samples was extracted using the TRIzol reagent (#15596018CN, Invitrogen) according to the manufacturer’s instructions. Then, the MeRIP assay was performed using Magna MeRIP m6A Kit (#17-10499, Millipore). The obtained purified RNA was decomposed into about 100-nucleotide-long oligonucleotides in the Fragmentation Buffer. After the fragment was finished, the lysate and antibodies anti-m6A (#ab208577, Abcam) were incubated overnight at 4 °C. These fragments were then reverse-transcribed before the 3′ adapters were ligated to the final cDNA. Only those fragments containing m6A modification were incorporated in the reverse-transcribed, which could be further detected by qPCR. All primers used in this study were summarized in Table S1.

### MS2 pull down

MS2bp-MS2bs-based pull-down assay was carried out to detect the direct interaction between the mRNA of CDKN1A and different proteins including POP1, YTHDF2, and RIDA. In short, pcDNA3-Flag-MS2bp and pcDNA3-12× MS2bs coupled with target RNAs or pcDNA3-12× MS2bs mock were transfected together into 5 × 10^6^ 293T cells. After 48 h, the cells were digested and then lysed in the lysis buffer (20 mM tris–Cl, pH 8.0, 0.5% NP-40, 1 mM EDTA, 10 mM NaCl) added with RNasin (#N2111, Promega). The obtained lysates were incubated with anti-Flag M2 affinity gel (#A2220, Sigma-Aldrich) and washed 5 times with the lysis buffer. The obtained proteins were used for Western blotting analysis.

### RNA stability assay

TNBC cells were treated with 5 μg/ml Act-D (#HY-17559, MedChemExpress) to inhibit gene transcription for all times. Then, RNA was extracted and quantified by qPCR at 0, 1, 2, and 3 h after treatment (normalized to the GAPDH expression). Subsequently, according to the formula *T*_1/2_ = ln2/*k*_decay_ adopted by Huang et al. [49], CDKN1A mRNA degradation curves of each group were fitted and the half-life of each group was calculated separately.

### Xenograft tumor model

Female BALB/c nude mice (4 to 5 weeks of age) were raised in a 12-hour light–dark cycle specific pathogen-free barrier facility. They were randomly grouped according to the experimental grouping requirements and fed under the same conditions. All procedures involving animals were conducted in line with the *Guide for the Care and Use of Laboratory Animals* and the institutional ethical guidelines of Sun Yat-sen University for animal experiments. All animal experiments were approved by the Ethics Committee of SYSUCC. In the experiment of subcutaneous tumor formation, MDA-MB-231 cells in each group were inoculated into the subcutaneous area of the mammary gland of mice (1 × 10^6^ each). The tumor size was measured with vernier calipers every 5 d, and the tumor volume was calculated. After 5 weeks, they were killed and tumor tissue was harvested. After the samples were photographed and weighed, they were fixed and paraffin embedded, tissue sections were prepared, and hematoxylin and eosin (H&E) and IHC staining were performed.

### Statistical analysis

We used R software (version 4.2.1) and GraphPad Prism (version 9.5.0) for statistical analysis and visualization of statistical results. Statistical analysis was conducted by 2-tailed Student’s *t* tests, one-way analysis of variance (ANOVA), Welch one-way ANOVA test, 2-way ANOVA test, log-rank, chi-square, and other methods when appropriate. Some statistical methods were indicated in the corresponding positions in the figure legend. Cox regression model was used for univariate and multivariate statistical analysis. *P* < 0.05 was considered statistically significant. Significant differences were shown by *P < 0.05, **P < 0.01, ***P < 0.001, and ns, not significant.

## Data Availability

All datasets referred to in this study can be obtained in online repositories referring to Materials and Methods. The raw data used for the mapping are available in Supplementary Raw_Data. All further inquiries can be directed to the corresponding author.

## Supplementary Materials

Supplementary 1 Figs. S1 to S6 Tables S1 and S2 Supplementary Raw_Data
