# Supplementary material for: POP1 Facilitates Proliferation in Triple-Negative Breast Cancer via m6A-Dependent Degradation of CDKN1A mRNA
Source: Research (Wash D C). 2024 Sep 12;7:0472. doi: 10.34133/research.0472 (PMC11391272; doi:10.34133/research.0472)
Supplement: Supplementary 1 — Figs. S1 to S6 Tables S1 and S2 Supplementary Raw_Data [file research.0472.f1.zip › Supplementary Figure.docx]

Supplementary Material

Supplementary Figures:


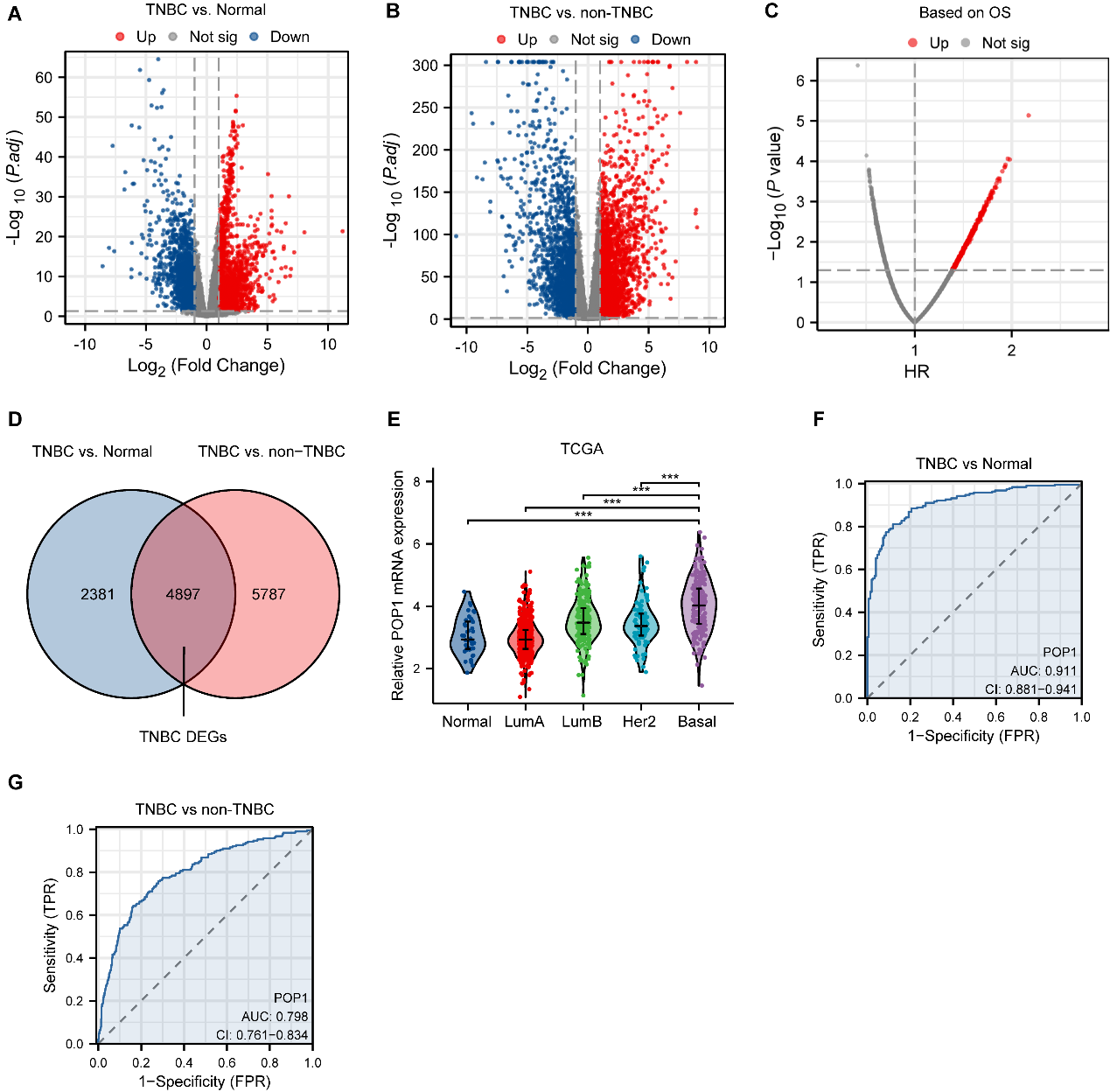


**Supplementary FigureS1.** **(A)** A volcanic map to obtain differentially expressed genes in TNBC versus normal tissues. **(B)** A volcanic map to obtain differentially expressed genes in TNBC versus non-TNBC tissues. **(C)** A volcanic map to obtain genes associated with poor prognosis in TNBC. **(D)** A Venn diagram to obtain the specific differentially expressed genes in TNBC. **(E)** The mRNA expression levels of POP1 in different types of breast cancer tissues and normal breast tissues based on the TCGA data. **(F-G)** ROC curves showed the diagnostic efficacy of POP1 expression in TNBC. ****P* < 0.001.


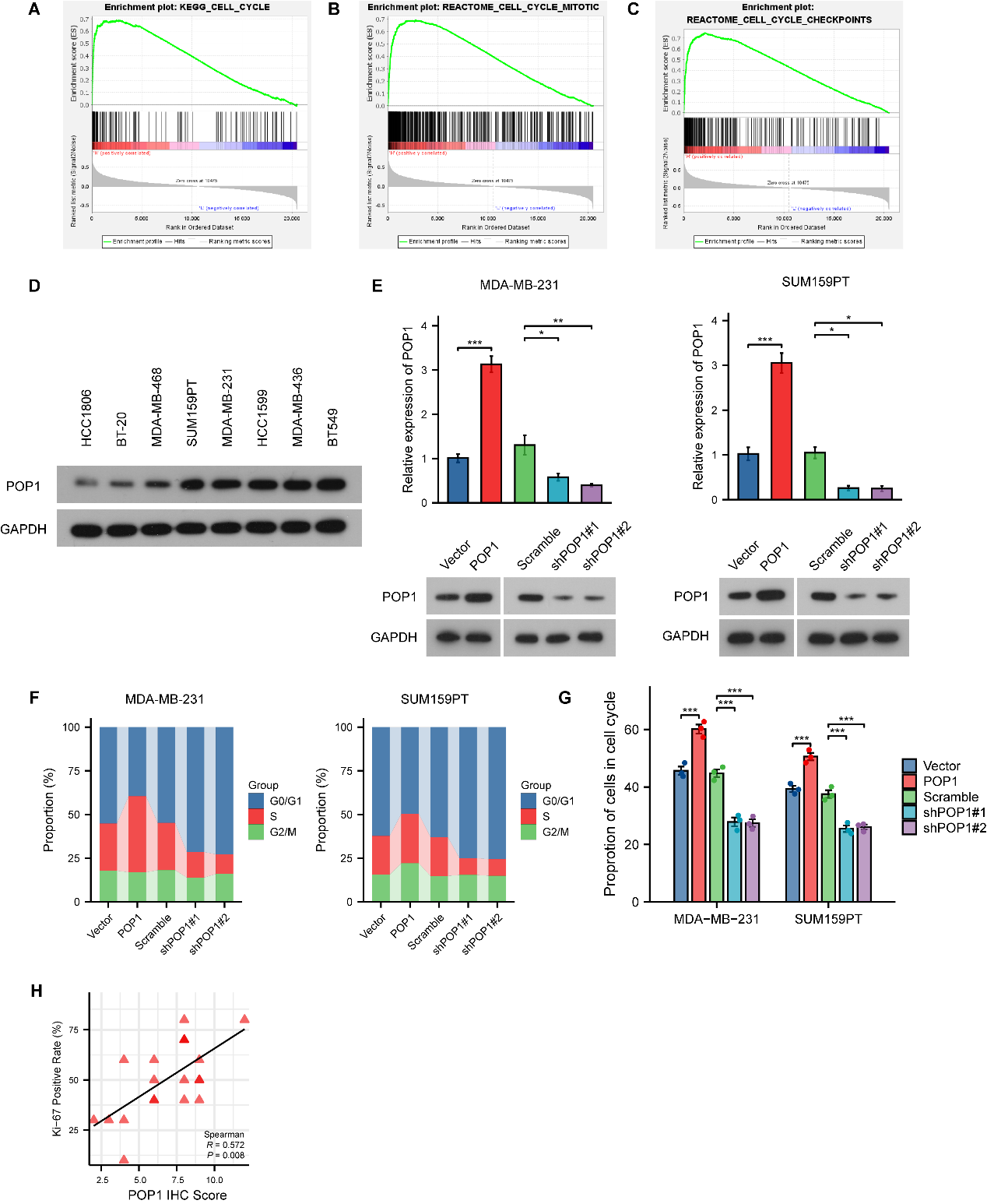


**Supplementary FigureS2.** **(A-C)** Three GSEA enrichment analysis results suggesting that high expression of POP1 was associated with cell cycle and mitosis. **(D)** Western blot analysis was performed to detect POP1 expression in 8 common TNBC cells. **(E)** The stable overexpression and knockdown cell models of POP1 were measured from mRNA and protein levels. **(F)** Cell cycle distribution of cells with different POP1 expression levels. **(G)** Comparison of the proportion of cells in proliferative state with different POP1 expression levels. **(H)** A scatter plot of correlation between POP1 immunohistochemical score and Ki-67 positive rate. **P* < 0.05, ***P* < 0.01, ****P* < 0.001.


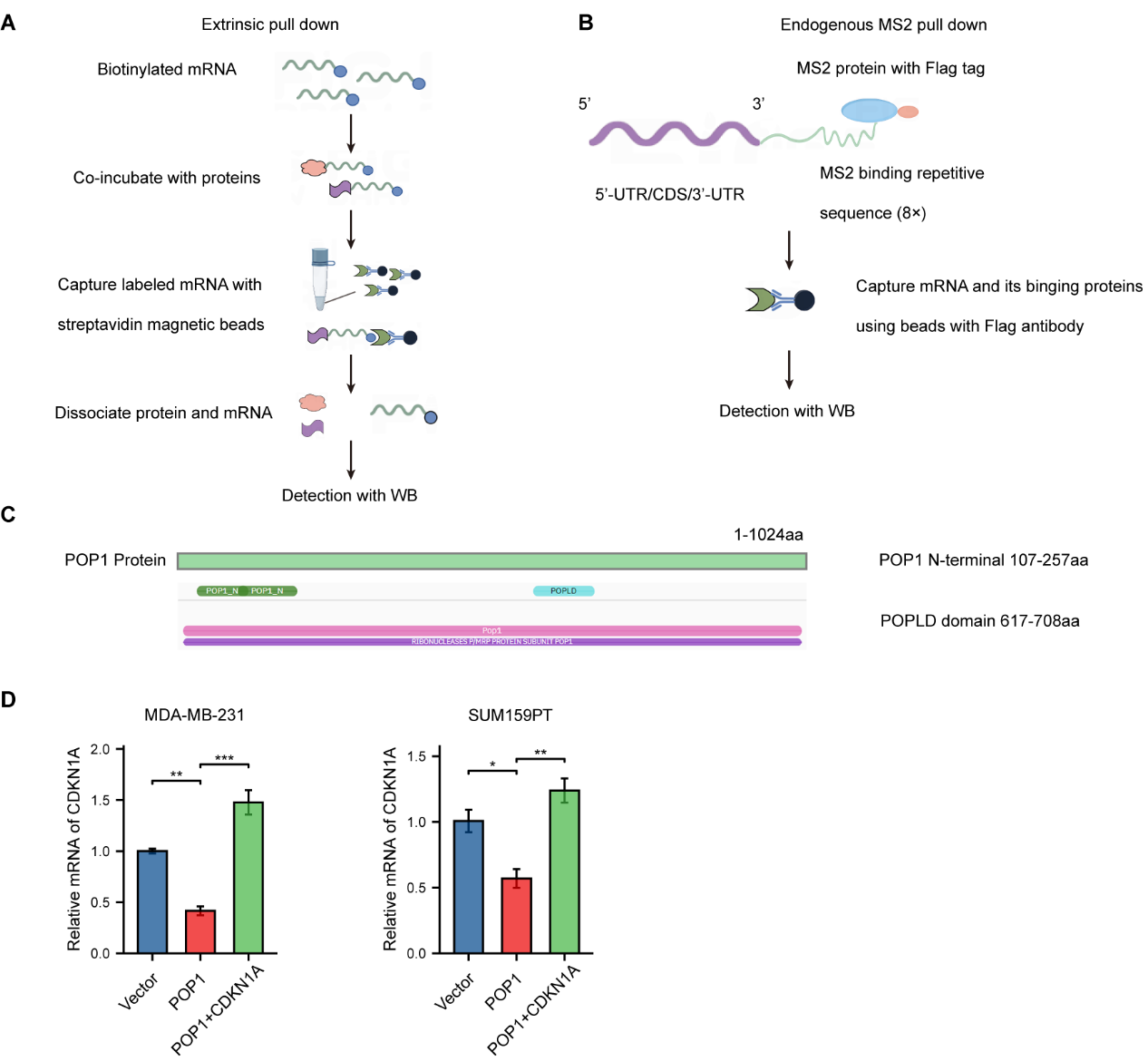


**Supplementary FigureS3.** **(A)** Schematic diagram of extrinsic RNA pull-down experiment. **(B)** Schematic diagram of endogenous MS2 pull-down experiment. **(C)** Domain information of POP1 reported so far. **(D)** Intracellular CDKN1A mRNA level after over-expression of CDKN1A. **P* < 0.05, ***P* < 0.01, ****P* < 0.001.


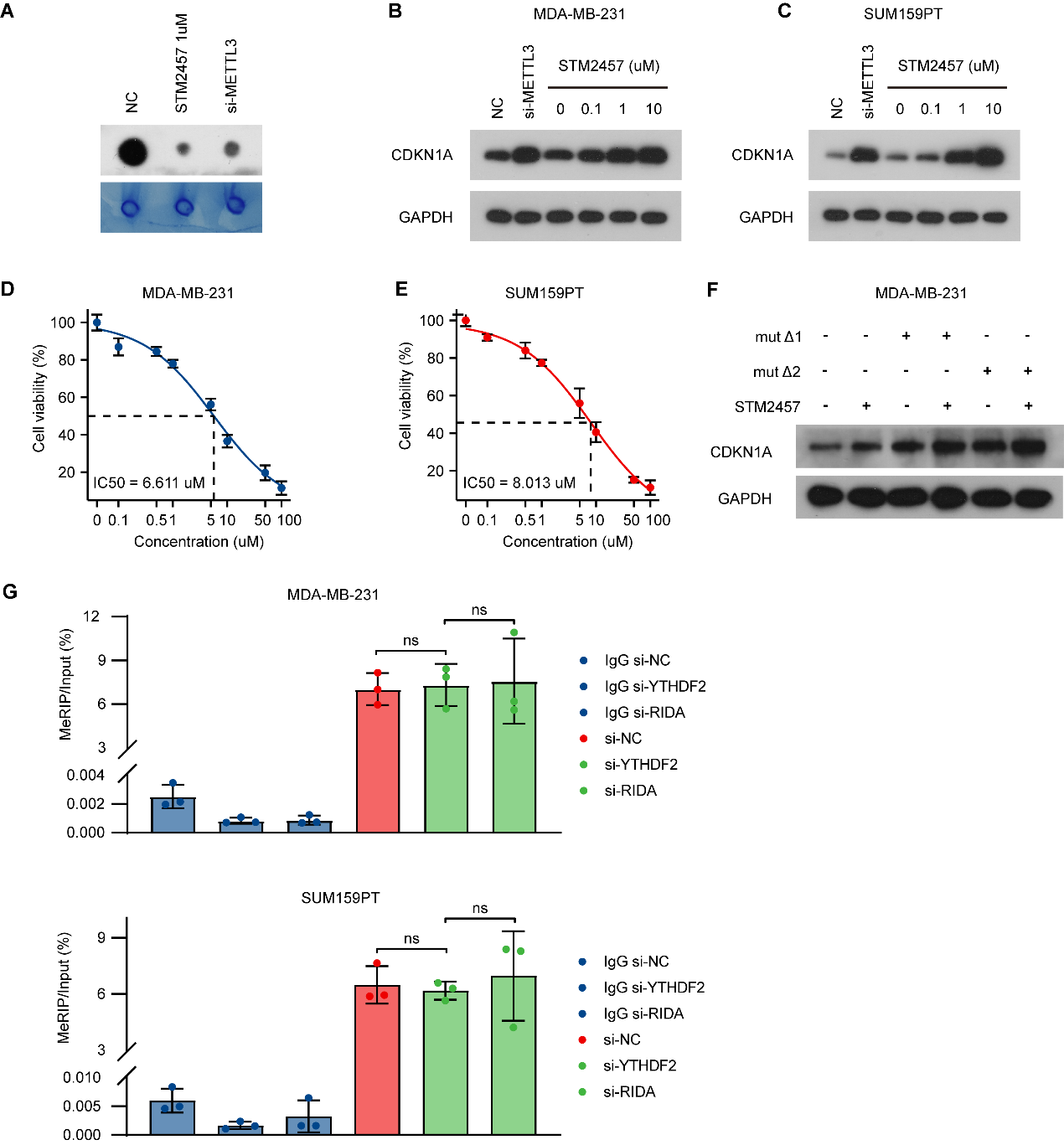


**Supplementary FigureS4.** **(A)** Dot blot assay was used to detect the effects of STM2457 treatment and si-METTL3 on the intracellular total m6A level. **(B-C)** Effects of different concentrations of STM2457 on intracellular CDKN1A expression. **(D-E)** IC50 determination of MDA-MB-231 and SUM159PT under STM2457 treatment. **(F)** Western blot to detect the effect of STM2457 on mutant CDKN1A. **(G)** M6A level of CDKN1A under different treatments in MDA-MB-231 and SUM159PT. ns, not significant.


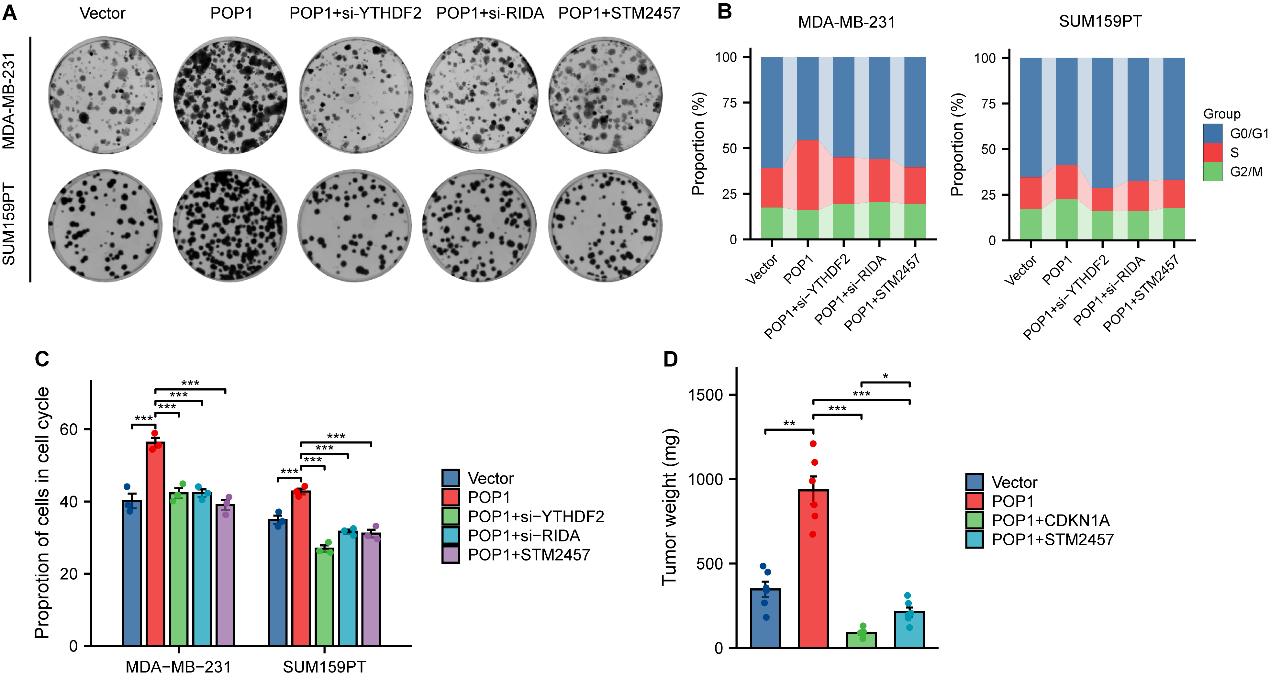


**Supplementary FigureS5.** **(A)** Representative results of colony formation assay of cells from different groups. **(B)** Cell cycle distribution of different cell groups in the rescue experiment. **(C)** Comparison of the proliferative proportion of cells in different groups in the rescue experiment. **(D)** Statistics of tumor weight of different groups in the tumor formation assay. **P* < 0.05, ***P* < 0.01, ****P* < 0.001.


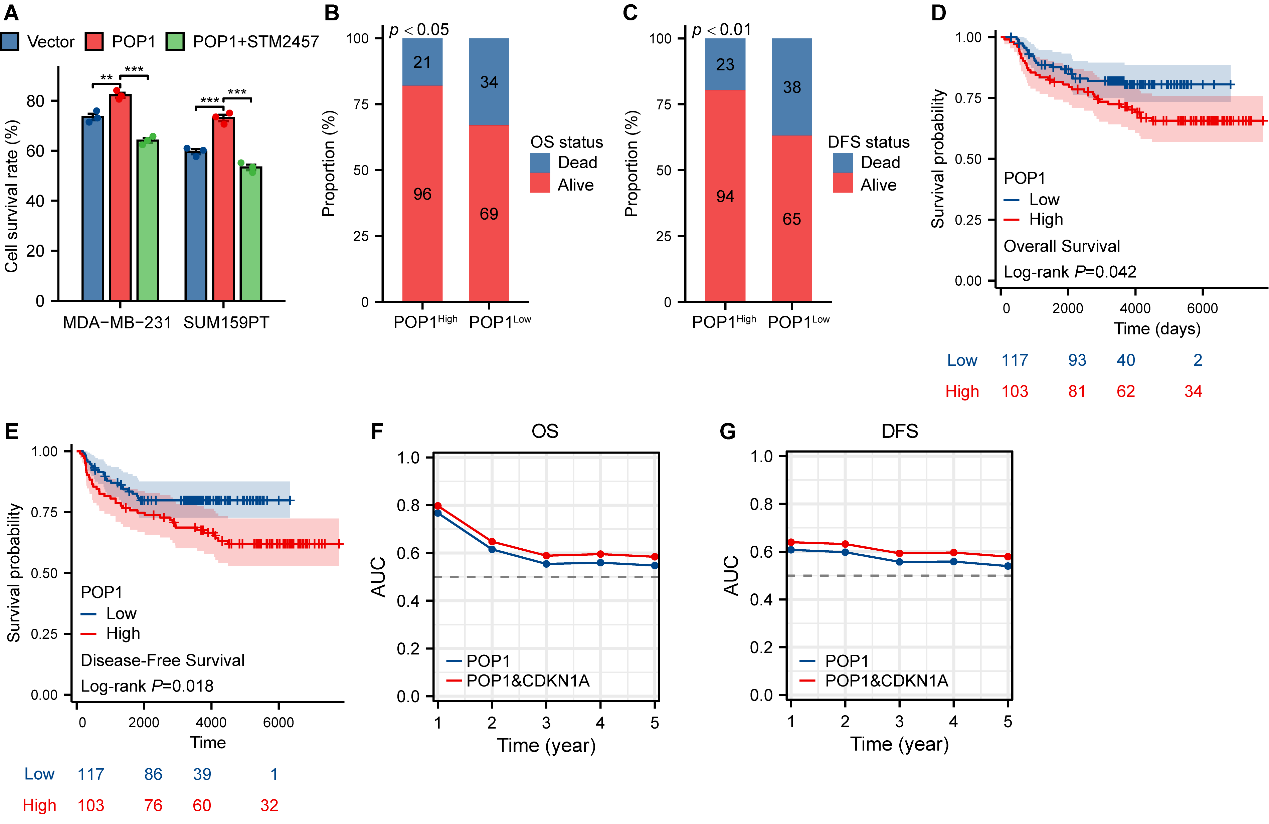


**Supplementary FigureS6.** **(A)** Cell survival statistics of different groups in the AnnexinV-PI flow cytometry assay. The proportion of POP1 expression in different OS **(B)** and DFS **(C)** outcomes. Survival curves of patients with different levels of POP1 expression included OS **(D)** and DFS **(E)**. **(F-G)** Time-dependent AUC curves showed the predictive efficiency of POP1 and CDKN1A alone or in combination for OS and DFS. ***P* < 0.01, ****P* < 0.001.
