# Supplementary figures and images for: POP1 Facilitates Proliferation in Triple-Negative Breast Cancer via m6A-Dependent Degradation of CDKN1A mRNA
Source: Research (Wash D C). 2024 Sep 12;7:0472. doi: 10.34133/research.0472 (PMC11391272; doi:10.34133/research.0472)

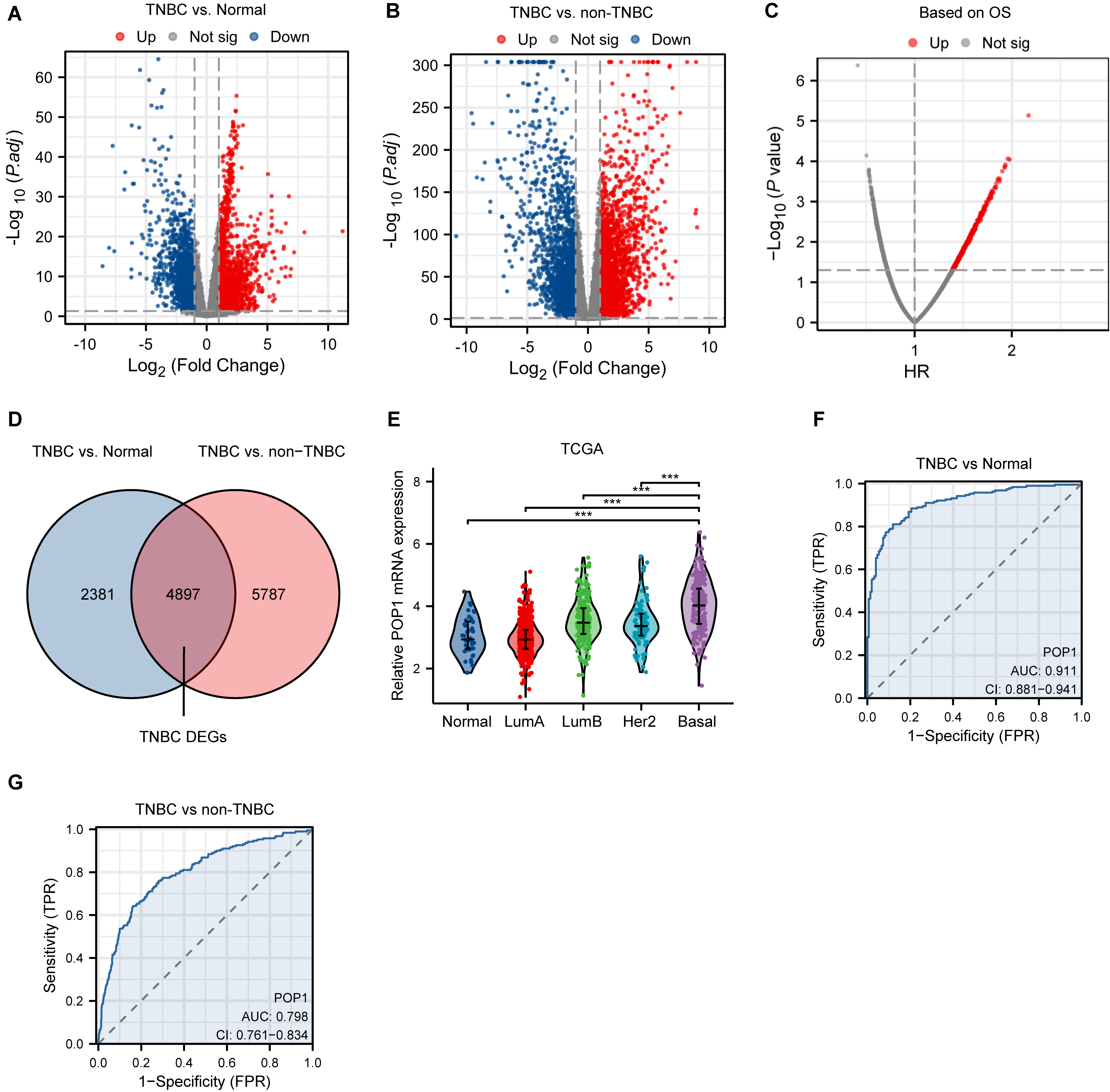

Supplement: Supplementary 1 — Figs. S1 to S6 Tables S1 and S2 Supplementary Raw_Data [file research.0472.f1.zip › Figure S1.tif]

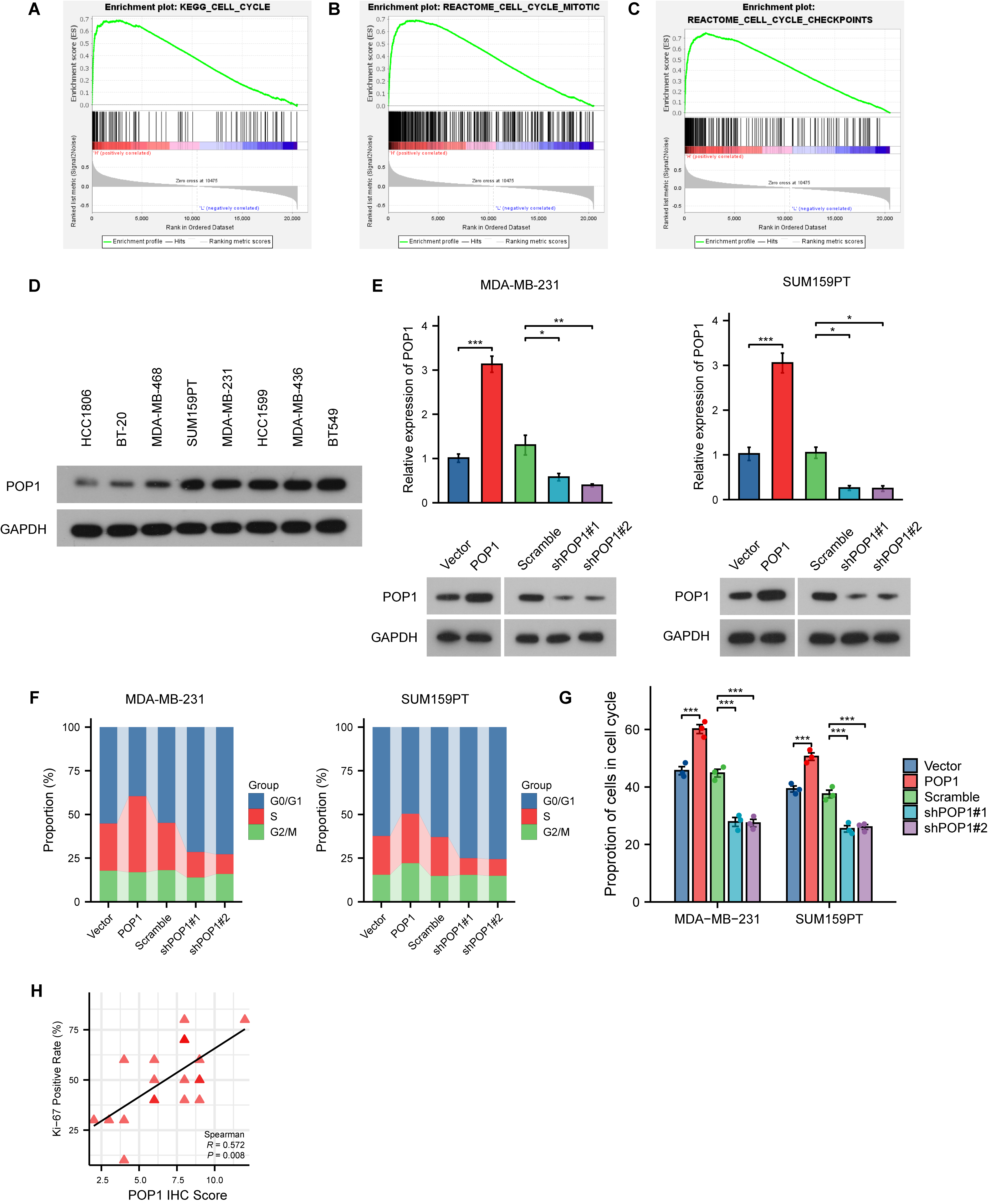

Supplement: Supplementary 1 — Figs. S1 to S6 Tables S1 and S2 Supplementary Raw_Data [file research.0472.f1.zip › Figure S2.tif]

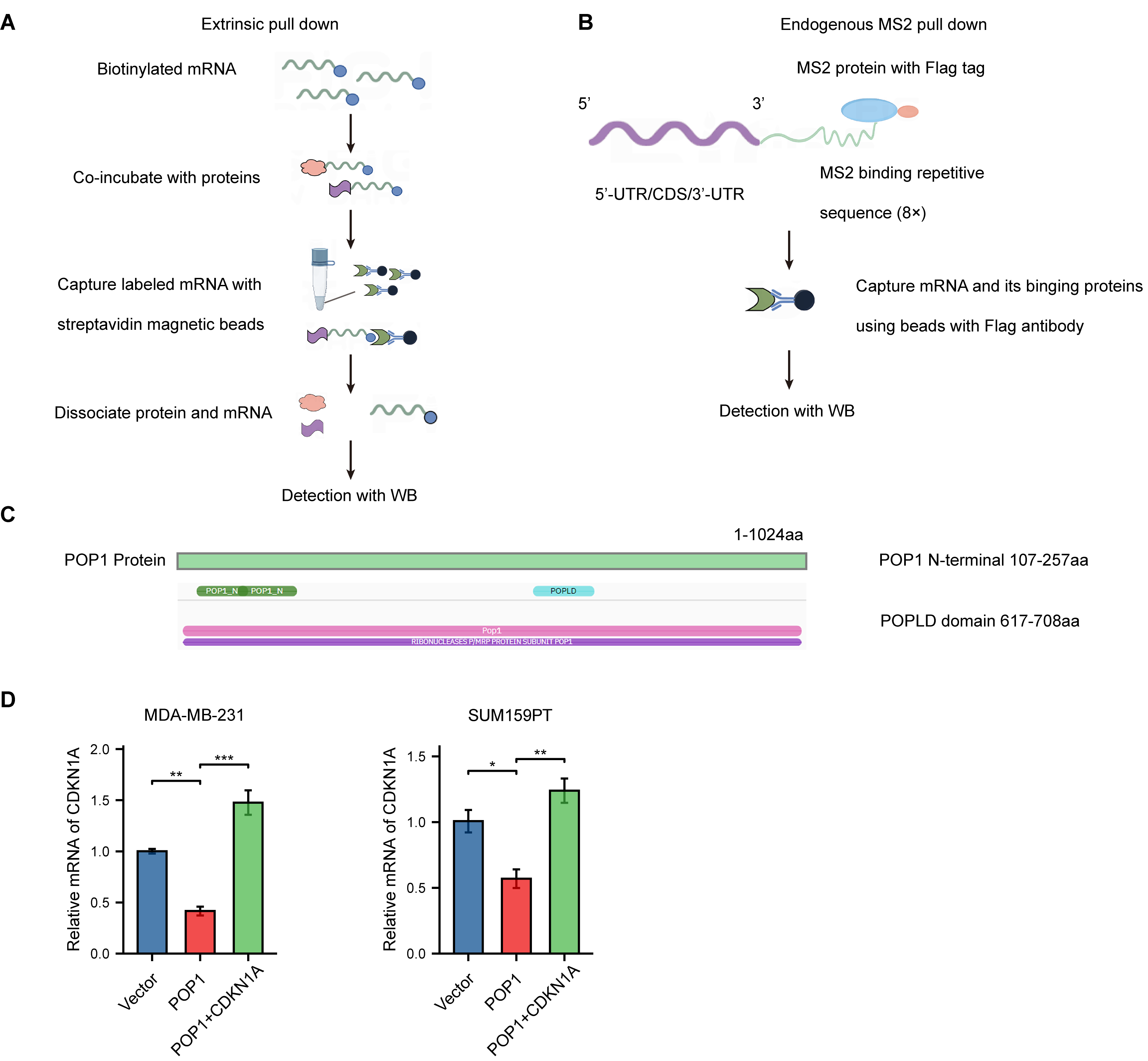

Supplement: Supplementary 1 — Figs. S1 to S6 Tables S1 and S2 Supplementary Raw_Data [file research.0472.f1.zip › Figure S3.tif]

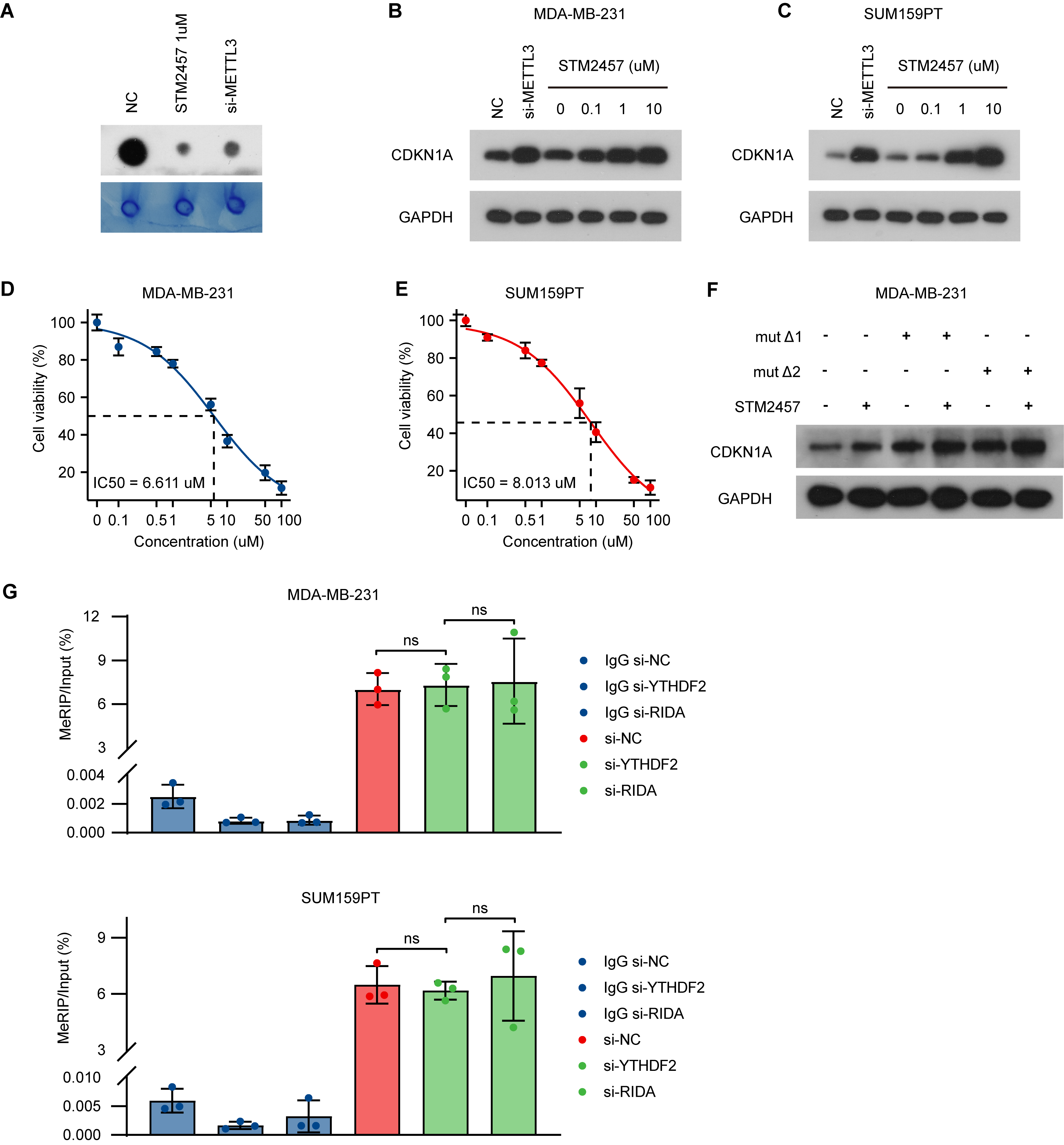

Supplement: Supplementary 1 — Figs. S1 to S6 Tables S1 and S2 Supplementary Raw_Data [file research.0472.f1.zip › Figure S4.tif]

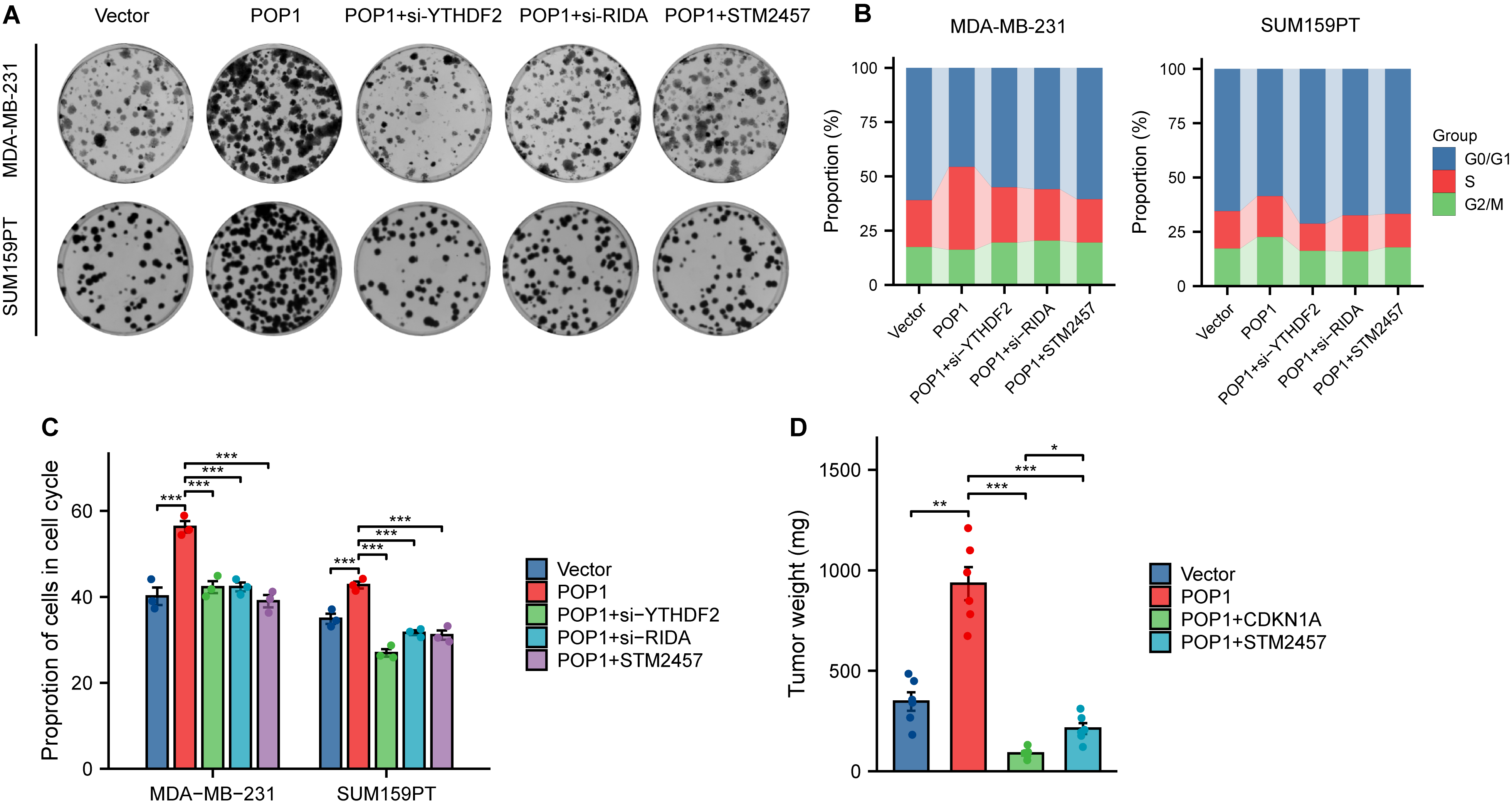

Supplement: Supplementary 1 — Figs. S1 to S6 Tables S1 and S2 Supplementary Raw_Data [file research.0472.f1.zip › Figure S5.tif]

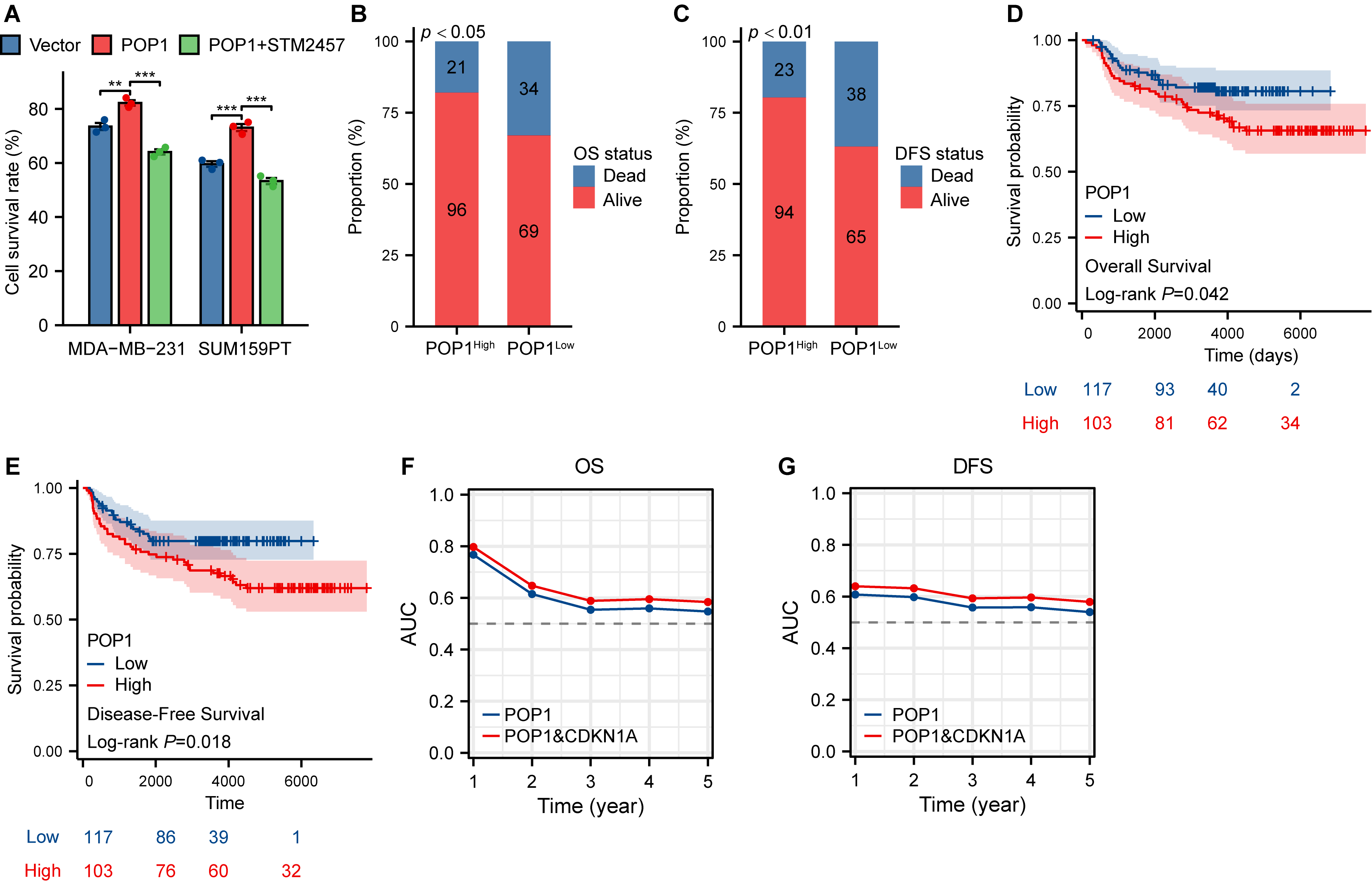

Supplement: Supplementary 1 — Figs. S1 to S6 Tables S1 and S2 Supplementary Raw_Data [file research.0472.f1.zip › Figure S6.tif]
